# A Scoping Insight on Potential Prophylactics, Vaccines and Therapeutic Weaponry for the Ongoing Novel Coronavirus (COVID-19) Pandemic- A Comprehensive Review

**DOI:** 10.3389/fphar.2020.590154

**Published:** 2021-02-26

**Authors:** Priyanka Dash, Subhashree Mohapatra, Sayantan Ghosh, Bismita Nayak

**Affiliations:** Immunology and Molecular Medicine Laboratory, Department of Life Science, National Institute of Technology Rourkela, Odisha, India

**Keywords:** COVID-19, SARS-CoV-2, vaccines, acute viral respiratory distress syndrome, epidemiology, pandemic

## Abstract

The emergence of highly virulent CoVs (SARS-CoV-2), the etiologic agent of novel ongoing “COVID-19” pandemics has been marked as an alarming case of pneumonia posing a large global healthcare crisis of unprecedented magnitude. Currently, the COVID-19 outbreak has fueled an international demand in the biomedical field for the mitigation of the fast-spreading illness, all through the urgent deployment of safe, effective, and rational therapeutic strategies along with epidemiological control. Confronted with such contagious respiratory distress, the global population has taken significant steps towards a more robust strategy of containment and quarantine to halt the total number of positive cases but such a strategy can only delay the spread. A substantial number of potential vaccine candidates are undergoing multiple clinical trials to combat COVID-19 disease, includes live-attenuated, inactivated, viral-vectored based, sub-unit vaccines, DNA, mRNA, peptide, adjuvant, plant, and nanoparticle-based vaccines. However, there are no licensed anti-COVID-19 drugs/therapies or vaccines that have proven to work as more effective therapeutic candidates in open-label clinical trial studies. To counteract the infection (SARS-CoV-2), many people are under prolonged treatment of many chemical drugs that inhibit the PLpro activity (Ribavirin), viral proteases (Lopinavir/Ritonavir), RdRp activity (Favipiravir, Remdesivir), viral membrane fusion (Umifenovir, Chloroquine phosphate (CQ), Hydroxychloroquine phosphate (HCQ), IL-6 overexpression (Tocilizumab, Siltuximab, Sarilumab). Mesenchymal Stem Cell therapy and Convalescent Plasma Therapy have emerged as a promising therapeutic strategy against SARS-CoV-2 virion. On the other hand, repurposing previously designed antiviral agents with tolerable safety profile and efficacy could be the only promising approach and fast response to the novel virion. In addition, research institutions and corporations have commenced the redesign of the available therapeutic strategy to manage the global crisis. Herein, we present succinct information on selected anti-COVID-19 therapeutic medications repurposed to combat SARS-CoV-2 infection. Finally, this review will provide exhaustive detail on recent prophylactic strategies and ongoing clinical trials to curb this deadly pandemic, outlining the major therapeutic areas for researchers to step in.

## Introduction

The world has confronted the global outbreak of several epidemics and pandemics caused by unknown coronaviruses (CoVs). Regardless of the immense advances in biomedical research, this century has been challenged with the frequent emergence of novel invading pathogens known to pose major and vulnerable alterations in the public healthcare system with a long-lasting dent in the global economy. In December 2019, the global human population experienced the drastic havoc of a deadly spillover i.e., coronavirus pandemics, elicited by the contagious SARS-CoV-2 pathogen (Cui et al., 2019). The clinical condition aroused by the phenotypically and genotypically diversified SARS-CoV-2 with the worldwide crisis has been officially announced as the current “COVID-19” threat by the World Health Organization (WHO) on March 11, 2020 (Chang et al., 2020); (Wu et al., 2020a). The first epicenter responsible for pneumonia of unknown etiology was the wholesale seafood market in Wuhan city, China (Phelan et al., 2020). Shortly, mounting infection statistics dramatically inflated with a high degree of fatalities in all nooks of the world. The incidence of ongoing COVID-19 pandemics is very rapid and violent, and from December 2019 to October 2020 has surpassed 72.7 million confirmed cases and more than 1.6 million fatalities.

Universally, mature SARS-CoVs (Nidovirales order) are the enveloped, positive-sense ((+)ss), and non-segmented RNA viruses with the largest linear genome of ∼32 kb (Zumla et al., 2016); (Banerjee et al., 2019). They are spherical virions with a diameter of around 100 nm. In general, a virus has a capsid, which acts as a shield to protect the viral genome. In addition, the protein capsid of SARS-CoVs is surrounded by lipids. The family Coronaviridae, are divided into α-CoVs, β-CoVs, δ-CoVs, and γ-CoVs genera (Al-Qahtani et al., 2020). SARS-CoV-2 is the world’s third-worst hit emerging β-CoVs following severe acute respiratory syndrome (SARS-CoV) and the Middle East respiratory syndrome (MERS-CoV) with a broad spectrum of disease severity, ranging from mild fever (88%), fatigue (40%), dry cough (69%) to severe, life-threatening multiple organ complicacies (conjunctivitis, encephalitis, etc), acute respiratory distress syndrome (ARDS), dyspnea, septic shock, tissue hypoperfusion and death (Yang et al., 2020b). As per clinical data, a patient’s especially the elderly suffering from diabetes, asthma, chronic coronary artery disease, hypertension, and so forth are highly susceptible to SARS-CoV-2 infection (Lovato and de Filippis, 2020); (Huang et al., 2020). The estimated median incubation period for COVID-19 is on an average between 2 and 14 days after natural exposure to SARS-CoV-2. The common mode of transmission of SARS-CoV-2 virion from person-person is primarily *via* respiratory droplets or through direct aerosolization of secretions (Banerjee et al., 2019); (Shanmugaraj et al., 2020); (Metcalf and Lessler, 2017); (Killerby et al., 2020). However, there is a pressing need to design novel and broad-spectrum anti-SARS-CoV-2 therapeutic medications not only to combat COVID-19 disease but also to counter the wide class of pre-existing resistant infectious virions and their mutants to rescue the global population from multiple life-threatening diseases.

**FIGURE 1:**
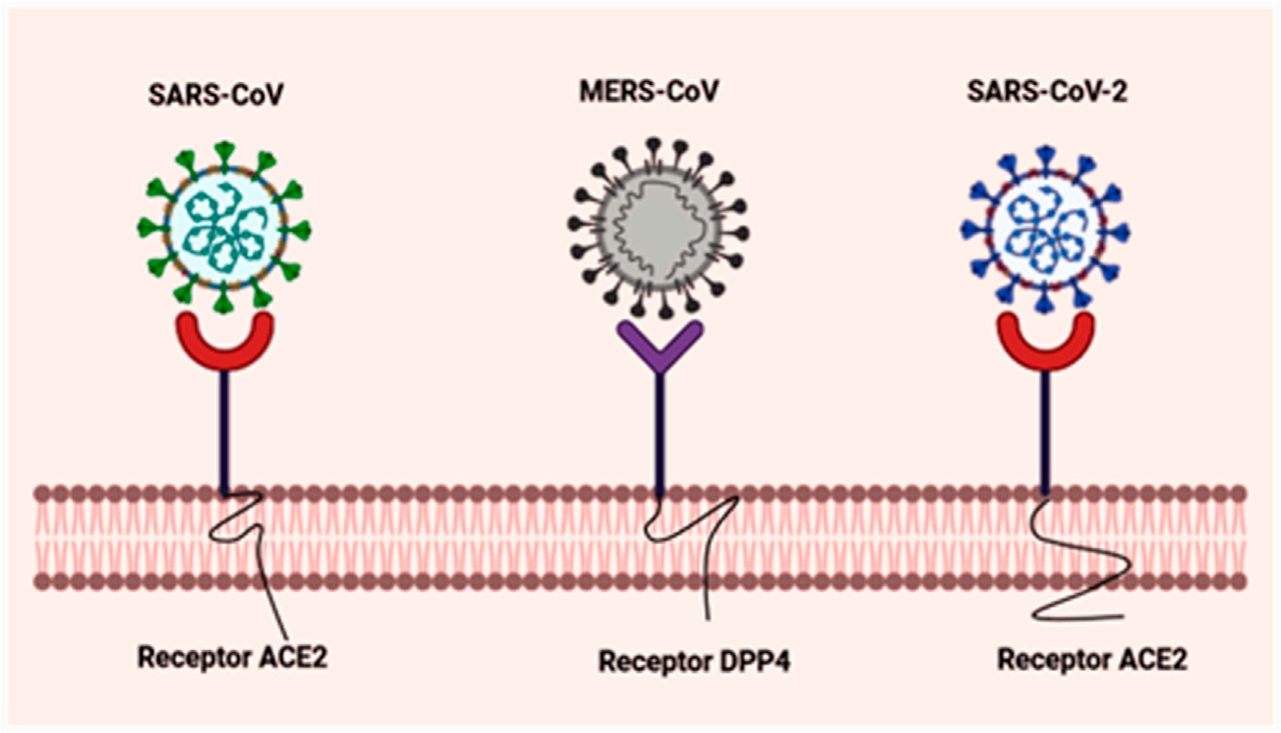
Schematic representation of molecular interaction between virus and host cellular receptors. The graphical representation portrays the S protein of SARS-CoV and MERS-CoV that interacts with the host cellular receptors ACE2 and DPP4, respectively. Novel SARS-CoV-2 also leverages human ACE2 as its target receptor for cellular entry, similar to SARS-CoV. Created with BioRender.com.

The causative agent of the COVID-19 pandemic shares a high degree of similarity with SARS-CoV in key genes, as evidenced *via* genomic sequencing as well as decade-long scientific analysis correlated to their proximal origin (Andersen et al., 2020). The whole RNA genome of SARS-CoV-2 encodes for structural (spike (S), nucleocapsid (N), matrix (M), and the envelope (E)) proteins, and non-structural proteins (Nsps) critical for its survival and virulence power. The nucleocapsid of β-CoVs is composed of a major structural phosphoprotein i.e., N protein-laden within phosphorylated lipid bilayers and is encased by two discordant S proteins; surface-exposed S glycoprotein trimmers which are present virtually in all SARS-CoVs and the hemagglutinin-esterases shared solely in some SARS-CoVs (Scotti and Scotti, 2020); (Forni et al., 2017); (Zhu et al., 2020b). The S protein is a glycosylated multifunctional molecular machine that promotes virion internalization into a target cell and is the sole viral membrane to determine viral tissue tropism and host range to some extent. The unique presence of these club-shaped peplomers or spikes on the surface of the virus, give SARS-CoV a crown-like appearance when viewed under a transmission electron microscopy (TEM) (Velavan and Meyer, 2020). S1 and S2 subunits of β-CoVs S protein play a crucial role in the recognition of cell surface receptor and membrane fusion, respectively. The former contains two functional RNA binding sub-domains: the C-terminal domain (CTD) and the N-terminal domain (NTD). The Receptor Binding Domain (RBD) of SARS-CoV implicated in recognition of host cell unique signatures (receptors) is localized in the CTD (Ou et al., 2017). But not all coronavirus RBDs are located in the CTD, the receptor of MHV (Murine Hepatitis virus) is CEACAM1 (not sugar), and its RBD is located in S1-NTD. NTD and CTD mediate the binding of the virion to sugar-based receptors, and the protein-based receptors, respectively (Tang et al., 2020). The M protein maintains the membrane structure of coronavirus whereas the E proteins contribute to the assembly and release of virions including their pathogenesis (Wu et al., 2020b). SARS-CoVgenetic makeup contains 5’ and 3′ terminal sequences, featuring a gene order 5‘–replicase open reading frame (ORF) 1ab-S-E-M-N-3‘ (Zhu et al., 2020b); (Lu et al., 2020). CTD of SARS-CoV-2 interacts with the metallopeptidase functional receptor named angiotensin-converting enzyme-2 (ACE2) to enter permissive cells, overall similar to SARS-CoV, whereas CTD of MERS-CoV binds dipeptidyl peptidase 4 (DPP4) receptor (Figure 1) (Wan et al., 2020). According to recent findings, the TMPRSS2 (serine protease-2) receptor also primes SARS-CoV-2 for entry into the target epithelial cells. These corresponding molecular interaction studies have been determined *via* the crystal structure of the protein complex (Wang et al., 2020c). The complex between SARS-CoV-2 and ACE2 forms the first line of infection, the most common therapeutic strategies aimed at blocking the key molecular target involves: 1) Blockade of SARS-CoV-2 spike by the administration of recombinant soluble ACE2, which plays the role of decoy receptors to trap the virus and, thus, to inactive virion internalization into the host cellular cytoplasm; 2) administration of specific antibodies by vaccination that specifically interacts with the S protein and blocks ACE2; 3) inhibition of host proteases to handicap SARS-CoV-2 and prevents its entry into the permissive cells (Weiss et al., 2020); (Dhama et al., 2020b). Accumulating clinical evidence on human tissue specimens has discovered the active ACE2 expression in the kidneys, epithelial cells of lungs, vascular endothelium, liver, small intestine, and the nasopharynx (Wan et al., 2020); (Hamming et al., 2004).

The development and design of excellent antiviral agents typically require a great deal of study in the context of scientific background (safety profile, efficacy, etc) before the medications are officially marketed. Furthermore, specific molecular targets of the virus might change or acquire resistance to the existing vaccines and drugs, as SARS-CoV-2 continues to mutate.

Confronted with such a pandemic, the present century highlights the crucial demand for the discovery of neoteric therapeutic interventions to fight against the deadly pneumonia virus (SARS-CoV-2). In this scoping review, we will explicitly stress upon currently available antiviral measures in accordance with their pharmacological and therapeutic effect (Table 1). In particular, in this comprehensive review, we discussed the current understanding of how HLA genetic variation plays a crucial role in identifying SARS-CoV-2 patients at a high risk of infection. In addition, the emphasis has been given to the real-time development of vaccines along with the global response towards the ongoing clinical trials which in future endeavours will have the potential for prophylaxis of the novel viral pathogen.

**TABLE 1:** Targets, Mechanism of action, required dose, safety profile, and improvements of repurposed anti-SARS-CoV-2 drugs.

Name	Targets	Mechanism	Dose and usage	Limitations	Improvements
Favipiravir	RdRp	Targets RdRp and inhibits viral transcription and replication mechanism	Duration of treatment-5 days. 1,800 mg twice (day 1) followed by 800 mg twice (day 2–5).	Teratogenic in nature	Works effectively in the form of combination therapy with Chloroquine and Azithromycin
Remdesivir	RdRp	Nucleotide analog that halts viral genome replication	Corse of medicine-<10, 200 mg (day 1) and 100 mg for the following days *via* intravenous instillation.	Respiratory failure, organ impairment	Unknown
Lymecycline	Viral survival proteins	Restricts the production of viral proteins essential for survival	408 mg, 1–2 capsules twice a day	No side effects	Unknown
Telmisartan	ACE2 receptor	Blocks angiotensin receptor	80 mg/12 h	No registered side effects	Unknown
Lopinavir	3CL^pro^	Impedes 3CL^pro^ activity	250 mg/50 mg capsule, oral intake of two capsules at a time twice per day	Toxic in nature, harmful impact on the immune system	Unknown
Chloroquine	Endosomal vesicles	Increases endosomal pH beyond normal	600 mg twice daily for 10 days	Diarrhea, alopecia, and loss of appetite	No concrete data of clinical effectiveness
Type I Interferon (IFN)	Viral replication	Suppresses viral replication in the host	450 mg, 1–2 capsules twice a day	No side effects	Works effectively in combination with other antiviral drugs
Plasma therapy	SARS-CoV-2	Virus neutralization	200 ml of plasma per day	Transfusion-associated reactions	Storage and distribution can be improved
Arbidol	Viral-cell membrane fusion	Inhibits host cell adhesion	200 mg, 1–2 capsules twice a day	No side effects	Unknown
Azithromycin	Translation of viral RNA	Inhibits protein synthesis	500 mg orally (Day 1), followed by 250 mg once a day (Day 2–5)	Abnormal heart rhythms, liver problems, myasthenia gravis	More effective with combined doses of Azithromycin and placebo
Baloxavir marboxil	Viral replicase enzymes	Inhibits crucial viral polymerase needed for replication	40 mg (wt < 40) single dose 80 mg (wt < 80) single dose	Fever, chills, muscle aches, sore throat, runny or stuffy nose	Unknown

## The Origin of Novel SARS-CoV-2

It is urgent to shed new light on the evolution and pathogenicity of the causative agent of an epidemic to be able to execute appropriate therapeutic measures and curb the trauma related to future global outbreaks. As the natural origin of the mysterious virion, SARS-CoV-2 remains unknown so, many theories have been extracted using its counterpart, the whole-genome level of SARS-CoV, which shortly may accumulate a robust understanding of the pathogenesis and biology of SARS-CoV-2 at a molecular level and assist in the design and synthesis of novel recombinant therapeutic measures to defeat COVID-19.

In 2002, Southern China witnessed the emergence of an unprecedented global threat caused by SARS-CoV (Fidler and Fidler, 2004). After the outbreak of SARS-CoV, genomic investigations and phylogenetic analyses indicated bats to be the probable natural reservoirs for all mammalian SARS-CoV, and the masked palm civet (small carnivore) was a potential mutated intermediate host responsible for SARS-CoV spillover to humans (Cui et al., 2019); (Zheng, 2020). Bats were also speculated to be the natural reservoir of MERS-CoV and dromedary camels were identified to be the intermediate host (Table 2) (De Wit and Munster, 2013).

**TABLE 2:** Comparison between SARS-CoV-2, MERS-CoV, and SARS-CoV.

	SARS-CoV-2	MERS-CoV	SARS-CoV
Disease	COVID-19	MERS	SARS
Pandemic Year	2019	2012	2002
Genetic material	ssRNA	ssRNA	ssRNA
Disease transmission	•Respiratory droplets	•Respiratory droplets •Camel milk ingestion	•Respiratory droplets •Cough and sneeze •Fecal-oral
	•Cough and sneeze		
	•Close contact with a patient		
	•Aerosol		
CoVs subfamily	Coronaviridae	Coronaviridae	Coronaviridae
Genus	β-coronavirus	β-coronavirus	β-coronavirus
Natural reservoir	Bats	Bats	Bats
Reproductive number	3.28	<1	3
Intermediate host	*Paradoxurus Hermaphrodites*	*Camelus dromedaries*	*Manis javanica*
Origin	Wuhan, China	Saudi Arabia	Guangdong province, China
Host receptors	ACE2 and TMPRSS2	DPP4	ACE2
Symptoms	•Myalgia •Rhinorrhoea •Headache •Chest pain •Anosmia	•Fever •Cough •Dyspnea	•Fever •Dry cough •Muscle spasm •Headache •Dyspnea •Diarrhea
Mortality rate	4%	35%	11%
Diagnosis	RT-PCR	PCR	PCR and antibody test
Treatment	Antiviral polytherapy	No specific treatment	Antiviral polytherapy
Size of the genome	26–32 kb	30 kb	29.7 kb

Genomic sequencing revealed that SARS-CoV-2 shares 96% nucleotide sequence homology with the BatCoV RaTG13 strain, which was isolated from a bat, *Rhinolophus affinis *from China’s Yunnan Province (2013), proving the fact that bats acted as natural reservoirs (Jaimes et al., 2020). Furthermore, the occurrence of Malayan pangolins (*Manis javanica*) served as the intermediate host which was investigated by Guangdong and Guangxi customs during the anti-smuggling operations. The Pangolin-CoV genome showed an 85.5–92.4% resemblance to SARS-CoV-2. Surprisingly, RBD of Pangolin-CoV genomes have 99% amino acid identical to SARS-CoV-2. Strikingly, the Bat CoV RaTG13 and SARS-CoV-2 shared only 89.2% amino acid identity in the RBD region (Tiwari et al., 2020). Till now, pangolin and bats have emerged as the only mammals identified to be highly affected by SARS-CoV-2 related SARS-CoV (Lam et al., 2020); (Li et al., 2020).

## Investigational Approaches and Novel Therapeutic Modalities for ANTI-COVID-19

### Potential Molecular Targets of COVID-19 for Drug Discovery

The preceding overview of the origin, virology, and general antiviral mechanism of SARS-CoV-2, laid the potential foundation to revitalize the innovative discovery of specific drugs and therapeutic measures. The common strategy to mitigate virion docking into a host cell is to actively target viral key elements i.e., RBD (center for therapeutic approaches) of S protein (inhibit entry of virion into target cells). Recently, Elfiky et al predicted the SARS-CoV-2 S protein-binding site with Glucose Regulated Protein 78 (GRP78), a cell surface signaling receptor. Their molecular docking perspective revealed that there is favorable binding between the III and IV region of the S protein model and GRP78. Region IV serves as the major traction force for GRP78 binding. Moreover, these nine residues can be clinically valuable to develop *de novo *therapies effective against SARS-CoV-2 (Ibrahim et al., 2020).

Nsps are known to mediate multiple mechanisms needed for viral replication and transcription machinery including host-virus interaction. For SARS-CoV-2, ORF1a/b encodes two co-terminal replicase poly-proteins- pp1a and pp1ab. These replicase poly-proteins are subsequently cleaved by proteases to form individual Nsps. Nsps3 and Nsp5 encodes two coronaviral cysteine proteases, a papain-like protease (PLpro) and a chymotrypsin-like protease (3CL^pro^), respectively for producing remaining Nsps. Nsps are also involved in the synthesis of viral RNA, for example, Nsp12, also called RNA-dependent RNA Polymerase (RdRp) is a conserved protein among coronavirus responsible for virus transcription and replication complex. Nsp8 acts as a primer for Nsp 12-RdRp RNA synthesis whereas the Nsp7-Nsp8 complex increases the affinity of Nsp12 to bind RNA and precede transcription and replication machinery. Hence, clinically approved enzyme inhibitors targeting these replicase proteins may exert anti-COVID-19 activity *in vitro* for therapeutic development.

## Drugs/Nucleoside Analogs Associated Therapies for Inhibiting SARS-CoV-2 Infection in Clinical

**FIGURE 2:**
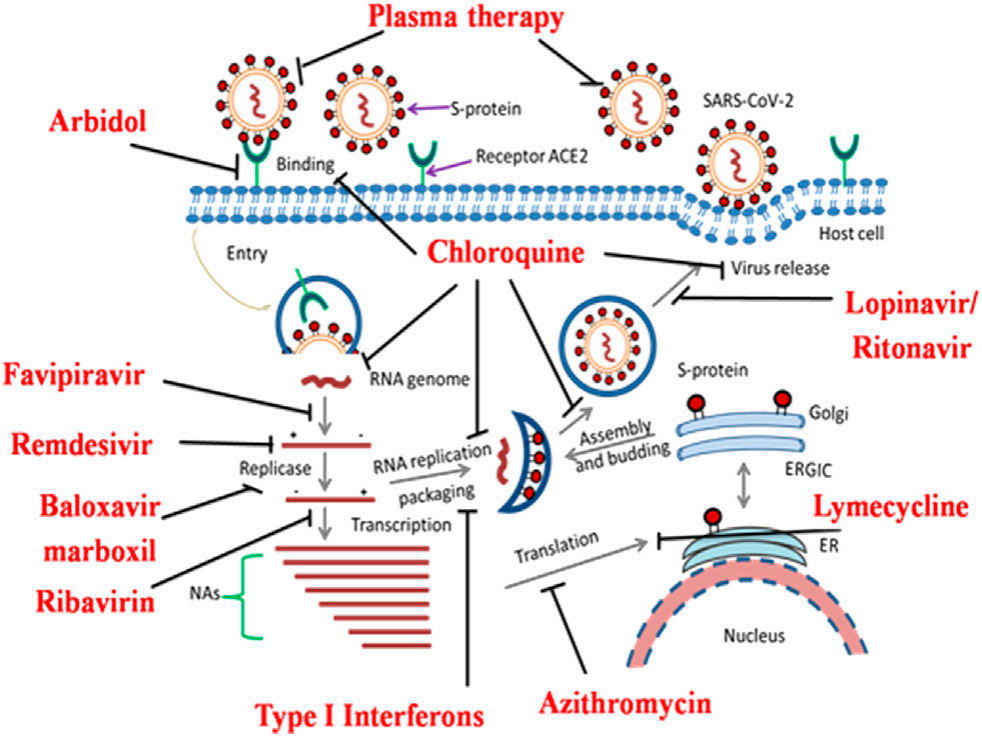
Overview of the novel SARS-CoV-2 pathogenesis and potential molecular targets for repurposed anti-COVID-19 drugs undergoing clinical trial studies. SARS-CoV-2 interacts with the host cellular receptor (ACE2). Later, virion particles gain entry into the target cells and undergo endocytosis. Inside the cell, due to low endosomal pH, the virus capsid disintegrates and the virion genome is released in the cytoplasm for protein synthesis mechanism. After the release of genomic RNA, it undergoes translation and replication forming sub-genomic mRNAs. The latter translates into a variety of structural (S, E, M, and N) proteins and accessory proteins in the endoplasmic reticulum (ER). RNA genome and a variety of cellular proteins undergo the formation of a new virus particle in the ER-Golgi intermediate compartment (ERGIC). Finally, the new virion particles are assembled and released *via* exocytosis for further pathogenesis. Re-proposed medications and their possible molecular targets against COVID-19 are depicted by bold lines.

To date, no effective anticoronavirus medications have been approved. However, there are large numbers of potential clinical studies taking place worldwide to examine the therapeutic capability of specific anti-viral drugs, vaccines, and antibodies based on the genomic and biophysical understanding of SARS-CoV. For example, determining key targets of SARS-CoV-2 may design small molecule inhibitors acting upon the functional proteins or enzymes associated with the virus replication cycle. Humanized monoclonal antibodies (mAbs) and fusion/peptide inhibitors may function as promising anti-COVID-19 drugs by targeting the S1 RBD and the S2 subunit, respectively *in vitro* or *in vivo* (Wang et al., 2020d); (Yuan et al., 2004). These target-dependent therapeutic strategies offer numerous options for the *de novo* discovery of anti-SARS-CoV candidates. Another strategy is also developed by boosting the human innate immune response, which plays a significant role against SARS-CoV. Maximum drugs developed to treat other infectious diseases are currently repurposed for clinical trials. Currently, clinical trials are specifically indulged in identifying combinatorial drug therapy that can be categorized into broad-spectrum specific host and virion based therapies. Nsps, a significant functional protein of SARS-CoV are ultimately involved in RNA replication, transcription, processing, protein synthesis, translation, membrane modification, and host infection. Among them, PLpro, 3CL^pro^, and RdRp are the most attractive and viable anti-SARS-CoV targets for the discovery of specific peptide inhibitors and small-molecule drugs to curb COVID-19. Here, we discuss in brief the ongoing therapeutic options that may successfully combat COVID-19 pneumonia (Figure 2).

## Inhibiting the RdRp

RdRp enzymes facilitate the replication and transcription of the viral RNA genome, including CoVs. As these essential enzymes are highly conserved across RNA viruses, thus are valuable targets for effective and safe antiviral therapies. Active prodrugs favipiravir and remdesivir inhibit viral RdRp enzymes and are now clinically validated for the treatment of SARS-CoV-2 infected patients.

### Favipiravir (T-705)

Favipiravir, otherwise marketed as “Avigan” has been designed as an oral anti-influenza compound by the Toyama Chemicals of Japan (Shiraki and Daikoku, 2020). The guanosine analogue favipiravir exhibits antiviral activity at a broader spectrum against multiple virulent RNA viruses, named influenza virus, rhinovirus, arenavirus, bunyavirus, ebola virus, and human orthopneumovirus both *in vitro* and *in vivo* (Furuta et al., 2002). However, favipiravir does not exert any lethal effect against DNA viruses. The antiviral mechanism of favipiravir acts by inhibition of vRNA polymerase (Furuta et al., 2017). Favipiravir directly targets the cleavage of RdRp blocking virion replication machinery and inhibiting infection. In contrast, Tamiflu, a potential inhibitor of neuraminidase blocks sialic acid (also called as neuraminic acid) catalytic site and the successive internalization of the virion into the host body. Unlike Tamiflu, favipiravir apparently lacks in the generation of resistant viruses (Shiraki and Daikoku, 2020). This best feature of favipiravir offers a novel therapeutic potential for treating the resurgence of SARS-CoV. However, the efficacy of favipiravir and interferon-α (IFN-α) (ChiCTR2000029600) composites have been tested on COVID-19 patients to evaluate the synergistic enhancement of host immune response and virion inhibition. In a multicentre randomized clinical trial study, avigan increased the recovery of COVID-19 infected subjects from 55.86 to 71.43% within seven days of treatment (Du and Chen, 2020). Indeed, favipiravir endorsed as the first anticoronaviral compound in China by the National Medical Products Administration of China (March 2020), as the controlled clinical trial study demonstrated no long-term toxicities with minimal known side effects. Recently, Wang *et. al* reported excellent *in vitro* antiviral potency of favipiravir towards SARS-CoV-2 with an EC_50_ value of 61 μM in Vero-E6 cells in a dose-dependent fashion (Cai et al., 2020). Favipiravir was combined with an antiviral agent, baloxavir marboxil to improve the healthcare complications of patients infected with COVID-19 pneumonia (Lou et al., 2020). Wan and coworkers have evaluated that high dose-response of favipiravir strongly decreases the virion load effect (Wang et al., 2020a). Pharmaceutical major Lupinon on August 6, 2020, launched favipiravir, under the brand name “Covihalt” (Rs 49 per tablet) to ameliorate the healthcare crisis of SARSCoV-2 infected patients. Moreover, drug giant Cipla gets a DCGI nod to successfully launch its convenient and cost-effective version of favipiravir, under the brand name “Ciplenza” in the country to treat COVID-19 patients. Glenmark pharmaceuticals also launched favipiravir, under the brand name “Faviflu” to treat mild to moderate cases of coronavirus. A Hyderabad-based-generic pharma MSN group launched “Favilo” as the cheapest drug to treat coronavirus-infected patients. Table 3 outlines the clinical trial studies for the validation of favipiravir’s safety profile.

**TABLE 3:** Running clinical trial studies for validation of Favipiravir (https://clinicaltrials.gov/ct 2/results).

Sl. No.	Title	NCT No.	Therapeutic Dose	Phase	Location
1	Safety and efficacy of Favipiravir in Management of COVID-19	NCT04349241	Loading dose: 1,600 mg (Every 12h), Day 2–10 (1,200 mg)	3	Egypt
2	Safety estimation of Favipiravir for COVID-19– management	NCT04336904	Loading dose: Day 1: 1,800 mg (BID) Days 2–14: 600 mg (TID)	3	Italy
3	Favipiravir efficacy in hospitalized SARS-CoV-2 patients	NCT04359615	Not specified	4	Iran
4	Oral injection of Favipiravir against mild infection	NCT04346628	Loading dose: 1,800 mg (Day 1 BID), Day 2–9 (800 mg BID)	2	U.S.
5	Favipiravir use in SARS-CoV-2 patients	NCT04358549	Loading dose: 1,800 mg (Day 1- twice a day), Day 2–14: 1,800 mg (twice a day)	2	U.S.

### Remdesivir

Remdesivir (GS-5734) is an antiviral medication, originally first concocted by the California-based biopharmaceutical company Gilead Sciences Inc. to thwart serious Ebola virus infection in the Democratic Republic of the Congo in 2014 (Mulangu et al., 2019). It is a promising prodrug of the nucleotide analog that mimics adenosine triphosphate (ATP) (Siegel et al., 2017). The antiviral strategy of remdesivir is a premature cessation of the nascent RNA chain by selectively inhibiting the RdRp enzyme and consequently perturbs the viral replication and transcription process. It also acts upon SARS-CoV infection by evading the proofreading by exoribonuclease enzyme (Warren et al., 2016). Remdesivir reflects the chemical structure of tenofovir alafenamide, a potent HIV reverse transcriptase inhibitor, and it exhibits antiviral efficacy at a broad-spectrum against diverse RNA viral pathogens, including β-CoVs, SARS-CoV and MERS-CoV *in vivo* (mice, non-human primates) and *in vitro* (Tchesnokov et al., 2019). A recently published report concerning the compassionate use of GS-5734 exhibited a positive clinical improvement in 68% of hospitalized COVID-19 patients, including 57% who were successfully extubated and recovered (Grein et al., 2020). Profoundly, it demonstrated minimal therapeutic effects during clinical trials against Ebola but proved to be safe for human use. Based on such an outstanding efficacy, recently remdesivir as a versatile therapeutic candidate has come into prominence for conduction of multiple clinical trials towards curbing the global emergency “COVID-19” (i.e., better the evidence, faster the survival). As per a clinical case report in the U.S. first COVID-19 patients recovered successfully with intravenous administration of remdesivir (Holshue et al., 2020), which indeed paves the way for remdesivir in the future to treat COVID-19 disease. As cases of SARS-CoV soar in the country, clinical trials on the therapeutic capability of remdesivir are being conducted worldwide, including two phase 3 randomized controlled trials (NCT04252664 and NCT04257656) in China. Interestingly, a recent investigation suggested that remdesivir and chloroquine phosphate combination exerts potential antiviral response against SARS-CoV-2 infection *in vitro* (Wang et al., 2020a)*;* (Liu et al., 2020c). Table 4 outlines clinical trial studies for the validation of remdesivir. As per scientific reports, drug firm Zydus Candela first announced the launch of the investigational drug, remdesivir under the brand name “Remdac” on August 14, 2020 to treat patients with COVID-19 symptoms at Rs 2,800 per 100 mg lyophilized injection in India. Cipla Ltd. and Hetero Healthcare Ltd. under the authorization of The United States Food and Drug Administration (USFDA) received DCGI approval for the successful launch of generic version of the remdesivir, under the brand names “Cipremi” and “Covifor”, respectively to cure emergency COVID-19 survivors. Mylan NV, a pharmaceutical major received DCGI approval towards successful launch of “Desrem”, generic brand of remdesivir in India at Rs 4,800 per 100 mg vial in the form of injections for treating coronavirus infected patients.

**TABLE 4:** Running clinical trial studies for validation of Remdesivir (https://clinicaltrials.gov/ct2/results).

Sl. No.	Title	NCT No.	Therapeutic Dose	Phase	Location
1	Clinical trial of COVID-19	NCT04280705	Loading dose: 200 mg (Day 1), Day 2–10: 100 mg (BID)	3	U.S.
2	Estimating the efficacy of various Anti-virals for SARS-CoV-2 infection	NCT04321616	Loading dose: 100 mg (Day 1–10)	2,3	Norway
3	Trial of treatments against SARS-CoV-2 infection in Adult hospitalized patients	NCT04315948	Loading dose: 100 mg (Day1) i.v. loading dose on the first day, 100 mg once daily	3	France
4	Determination of safety and efficacy of remdesivir against severe infection of SARS-CoV-2	NCT04292899	Loading dose: 200 mg (Day1), Day 2–5: 100 mg	3	U.S.
5	Clinical trial for Determination of the safety and Activity of remdesivir in patients	NCT04292730	loading dose: 200 mg on the first day followed by 100 mg on Day 2–5	3	U.S.

## Inhibiting the Viral Proteases

### Inhibiting PLpro

PLpro, a coronaviral protease cleaves NTD of the replicase poly-proteins to elicit vital Nsps essential for viral genome machinery (Harcourt et al., 2004). In addition, an investigation confirmed the significant antagonistic mechanism of PLpro on host innate immune response (Li et al., 2016); (Yuan et al., 2015). PLpro has been identified as an excellent drug target to combat COVID-19 disease due to its active involvement in the virus replication process. To date, no potent FDA approved inhibitor targeting PLpro has been marketed to cure SARS-CoV-2 infection.

Following the latest screening results of Chan et al. on potential inhibitors of PLpro extracted from the Zinc drug database, revealed that valganciclovir, ribavirin, and thymidine (anti-viral drugs), chlorphenesin carbamate (also known as Musil), a skeletal muscle relaxant drug exhibits high-affinity binding to PLpro (Chan, 2020). Naturally derived compounds targeting PLpro are *Platycodon grandifloras* derived platycodin D, *Cyperus rotundus *derived sugetriol-3,9-diacetate, *Scutellaria baicalensis *derived baicalin, and so forth (Wu et al., 2020a).

### Inhibiting 3CL^pro^

3CL^pro^, a cysteine protease also known as C30 endopeptidase specifically cleaves SARS-CoV-2 poly-proteins at 11 sites at CTD to elicit Nsps4 to Nsps16. 3CL^pro^ promotes the maturation of vital Nsps, necessary in the regulation of the virion life cycle. According to theoretical evidence, the lopinavir/ritonavir combination selectively inactivates the 3CL^pro^ protease of coronavirus (Sang et al., 2020). Currently, there is no human homologous for 3CL^pro^, which makes it an ideal broad-spectrum antiviral target.

Montelukast, an anti-asthmatic candidate is a well-researched drug having a high affinity for the 3CL^pro^ cleavage site. It was investigated to suppress oxidative stress. It has been hypothesized that the intake of high doses of montelukast has been beneficial in inflammatory disease (Asthma) (Fidan and Aydoğdu, 2020). As the elevation in mortality is due to the elicitation of excess inflammatory responses, hence in response to such adverse clinical conditions montelukast can be investigated further to limit the wild disease progression. As compared to placebo, currently investigation on this anti-allergic agent has reached phase III clinical trial (Chams et al., 2020).

Some other drugs having an affinity for 3CL^pro^ are lymecycline, demeclocycline, and doxycycline (antibacterial drugs), nicardipine, and telmisartan (antihypertensive drugs), and conivaptan, a non-peptide inhibitor of vasopressin treating hyponatremia, showed high binding affinity to 3CL^pro^.

### Lopinavir/Ritonavir

Lopinavir (ABT-378) is an antiviral medication that retains inhibitory activity against type I aspartyl protease in human immunodeficiency virus-1 (HIV-1) (Ito et al., 2020). Ritonavir in combination with lopinavir enhances (“boosts”) the plasma half-life, concentration, and antiviral mechanism of the latter by inhibiting cytochrome P450 and is therefore often used as combination therapy to help control HIV infection (Perry et al., 2005). The trade name of the protease inhibitor combination is Kaletra^TM^ (Tobaiqy et al., 2020), which possesses a broad spectrum *in vitro* anticoronaviral strategy against SARS-CoV and MERS-CoV *in vitro* (McKee et al., 2020). A clinical case study revealed that lopinavir may ameliorate COVID-19 complications (Liu et al., 2020b). Indeed, the lopinavir/ritonavir drug regimen progressively displayed good clinical outcomes in a COVID-19 patient in Korea by a synergistic reduction in viral load effect (Lim et al., 2020). A retrospective cohort study further supported that lopinavir monotherapy is an excellent medication to alleviate the spread of COVID-19 (Yao et al., 2020a). However, an open-label, randomized clinical trial study (NCT04252885) using a lopinavir/ritonavir-based regimen displayed no significant clinical improvement in coronavirus pneumonia patients (Cao et al., 2020). Similarly, Cao *et al.*, reported another randomized clinical trial (ChiCTR2000029308) executed on COVID-19 patients reported no significant improvement in clinical outcomes after administration of combination therapy (lopinavir/ritonavir) compared to standard of care (Cao et al., 2020). Subsequently, Baden and colleagues reported findings from a therapeutic study; the results suggested that the higher lopinavir/ritonavir concentration may be required to perturb SARS-CoV-2 RNA replication in the lungs compared to the serum level (Baden and Rubin, 2020). In addition, Yamamoto *et al.*, reported that nelfinavir (trade name: Viracept), a potent inhibitor of HIV protease, strongly inhibits the infection and replication of SARS-CoV, hence, suggesting an ideal therapeutic approach towards COVID-19 treatment (Yamamoto et al., 2004). The very first SARS-CoV-2 infected patient of Wuhan received 400 and 100 mg of lopinavir/ritonavir respectively on the fourth day of the infection. Gradually, her dyspnea improved with a reduced need for oxygen, and no traces of lung lesions were reported as observed from chest radiography (Yamamoto et al., 2004). A clinical study of lopinavir/ritonavir was conducted in 199 adult COVID-19 infected patients, in which 99 patients received polytherapy of lopinavir (400 mg) and ritonavir (100 mg) twice a day (BID) for about 4 days while rest 100 infected individuals were given standard care. The mortality rate of group which received polytherapy was observed to be 19.2% at 28 days and 25% in the standard care group (Cao et al., 2020). Table 5 outlines the clinical trial studies for the efficacy validation of Lopinavir/Ritonavir.

**TABLE 5:** Running clinical trial studies for validation of Lopinavir/Ritonavir (https://clinicaltrials.gov/ct 2/results).

Sl. No.	Title	NCT No.	Therapeutic Dose	Phase	Location
1	Evaluation of combinatory effect Lopinavir/Ritonavir, Ribavirin and IFN-β against SARS-CoV-2	NCT04276688	Loading Dose: 400 mg, and 100 mg (Lopinavir/Ritonavir Day 1–14) BID, 400 mg (Ribavirin Day 1 -14, 0.25 mg Interferon (Day 1–3)	2	HongKong
2	Evaluation of Traditional Chinese Medicines against SARS-CoV-2	NCT04251871	Loading dose: Lopinavir (400 mg), ritonavir (100 mg) twice a day (2 weeks).	Not applicable	China
3	Clinical trial of Lopinavir/Ritonavir for hospitalized COVID-19 patients	NCT04315948	Lopinavir/Ritonavir (200 mg). Lopinavir and ritonavir (50 mg) IFN-β-1a : 44 µg/0.5 ml (single dose)	3	France

### Ivermectin

Ivermectin, an antiparasitic FDA-approved medication belongs to the avermectin family; as these compounds are produced synthetically by *Streptomyces avermitilis* (soil microorganism) (CAMPBELL and BENZ, 1984). It was first commercially approved in 1981 for animal use (Sharun et al., 2019). Ivermectin has been proven to exert broad-spectrum antiviral (HIV, dengue virus, West Nile), anticancer, and antibacterial properties (Crump and Omura, 2011).

Ivermectin was found to exert *in vitro* inhibitory activity towards the flavivirus replication process by specifically targeting the non-structural-3 helicase (NS-3). It potently inhibits the yellow fever virus but is a weak inhibitor of Japanese encephalitis, tick-borne encephalitis virus (flavivirus) (Mastrangelo et al., 2012); (Sharun et al., 2020a). Interestingly, it dissociates importin [alpha]/[Beta]1 heterodimer, responsible for translocation HIV-1 integrase and Nsp5 polymerase of dengue virus across the nuclear pore complex (Wagstaff et al., 2012). As active importin [alpha]/[Beta]1- mediated nuclear import is a crucial step in the regulation of replication cycle of several RNA viruses i.e., the process of infection, hence, targeting the process of nuclear transport may be a novel and viable therapeutic strategy in blocking the nuclear trafficking of viral RNA proteins (Yang et al., 2020a). Ivermectin suppresses pseudorabies virus replication by inhibiting the nuclear import of UL42 (an accessory subunit of DNA polymerase) (Lv et al., 2018). A similar inhibition mechanism was reported for bovine herpesvirus (DNA virus) (Raza et al., 2020). Recently, Caly and coworkers efficiently proved ivermectin’s capability in suppressing the replication of SARS-CoV-2 up to ∼5,000-fold in Vero-hSLAM cells after 24–48 h of infection (Caly et al., 2020). As per hypothesis, synergistic combinatorial therapy of hydroxychloroquine (HCQ) and ivermectin may exert a potent antiviral effect on SARS-CoV-2. In this combination cocktail, HCQ inhibits viral entry into the host cells, whereas ivermectin acts as inhibitor of viral replication cycle (Patrì and Fabbrocini, 2020). The *in vitro* antiviral potential of ivermectin against SARS-CoV-2 virion has further extended the antiviral spectrum of this drug. In the coming days, considering the promising and positive result of the *in vitro* study with an established safety profile, validation of the antiviral potential of ivermectin is of utmost importance *in vivo* model for treating ill SARS-CoV-2 infected patients with adequate dosing and obtain an insight into the possible infection mechanism by further evaluating this wonder drug in randomized clinical control trials. Table 6 outlines the running clinical trial studies for validating efficacy of ivermectin.

**TABLE 6:** Running clinical trials for validation of Ivermectin (https://clinicaltrials.gov/ct2/results).

Sl. No.	Title	NCT No.	Therapeutic Dose	Phase	Location
1	Validation of Ivermectin and Nitazoxanide efficacy	NCT04351347	Not specified	2,3	Egypt
2	Validating polytherapy of Nitazoxanide and Ivermectin	NCT04360356	Not specified	2,3	Egypt
3	Combinatorial therapy of HCQ and Azithromycin along with Ivermectin	NCT04343092	Ivermectin (0.2 mg/kg, 2 tablets of 6 mg per week)	1	Iraq
4	Clinical trials for promoting safety and efficacy of Ivermectin or Endocrine therapy	NCT04374279	Ivermectin (600 µg/kg), once/day for 3 days	2	U.S.
5	Evaluation of Ivermectin along with standard regimen	NCT04373824	Ivermectin (200–400 mcg/kg) for first day and the second day was given with standard care	Not applicable	India

## Blocking the Virus-Cell Membrane Fusion

### Umifenovir (Arbidol Hydrochloride)

Umifenovir (trade name: Arbidol) is a potent hydrophobic indole-based antiviral medication for the prophylaxis of seasonal influenza. Umifenovir is a fusion inhibitor approved by Russia and China to target Influenza hemagglutinin (HA) glycoprotein (Kadam and Wilson, 2017). The antiviral mechanism of umifenovir acts by blocking the fusion process of virus membrane-free endosome after endocytosis (Boriskin et al., 2008). Currently, umifenovir has been prioritized with other antiviral medications in a few clinical trial studies to tackle an outbreak of COVID-19. Arbidol monotherapy is also undergoing clinical trials (NCT04260594, NCT04255017). An open-label, randomized control trial exhibited in China (ChiCTR2000030254) comparing the therapeutic effects of arbidol with favipiravir showed a significant superior safety profile of favipiravir over arbidol (Shiraki and Daikoku, 2020). In a case series study on COVID-19, polytherapy of arbidol, lopinavir/ritonavir, and Chinese-based traditional medication mitigated SARS-CoV-2 pneumonia symptoms in four patients with a subsequent reduction of virion load levels to undetectable in two patients (Wang et al., 2020e). A small retrospective study on SARS-CoV-2 infected patients (*n* = 33) compared combination therapy of umifenovir plus lopinavir/ritonavir with lopinavir/ritonavir monotherapy against SARS-CoV-2 infection for 5–21 days. The combination therapy showed faster viral clearance on days 7 and 14, with consequent improvement in chest CT images obtained on Day 7 (Deng et al., 2020). In contrast, in monotherapy higher doses of adrenocortical hormones usage delayed SARS-CoV-2 viral clearance, indicating monotherapy with arbidol is ineffective.

### Chloroquine Phosphate (CQ) and Hydroxychloroquine (HCQ)

CQ and HCQ, an anti-autoimmune, and antimalarial chemotherapeutic agents have been sporadically used for treating patients with malaria, Q fever, rheumatic arthritis, and some other intracellular bacterial infections like Whipple’s disease, etc. Importantly, these chemotherapeutic agents also exert a broad antivirus activity. HCQ (a derivative of CQ) obstructs viral genome replication, mature viral assembly, and release by elevating pH in the intracellular vesicles (endosome) required for viral internalization into the cell (Savarino et al., 2003). Meanwhile, it was confirmed to suppress SARS-CoV-2 replication by interfering with ACE2 glycosylation (Vincent et al., 2005). Both CQ and HCQ have been shown to induce a concentration-dependent *in vitro* anticoronavirus strategy against SARS-CoV-2 (Yao et al., 2020b). Indeed, CQ as an antiviral therapeutics reported being effective with a tolerable safety profile by the National Health Commission (NHC) of the People’s Republic of China for treating patients suffering from critical coronavirus disease (Gao et al., 2020).

Multiple clinical trial studies in China demonstrated the strong efficacy of the HCQ drug in overcoming diverse SARS-CoV-2 associated with clinical complications. Similarly, a non-randomized clinical trial conducted in France showed that polytherapy of HCQ plus azithromycin exhibited a positive effect towards tackling the SARS-CoV-2 associated healthcare crisis (Gautret et al., 2020). In keeping with this, HCQ medication gained special Emergency Use Authorization (EUA) from the FDA on June 15, 2020, for combating the ongoing pandemic (COVID-19) spread in the USA. It is noteworthy that recently no substantial evidence of rapid viral clearance was reported in SARS-CoV-2 infected patients when administered with HCQ and azithromycin polytherapy, hence, suggesting the urgent need of the hour for conducting more clinical trial studies (Davidescu et al., 2020). Keyaerts *et al.* showed *in vitro* inhibitory efficacy of CQ towards the SARS-CoV replication process (Keyaerts et al., 2004). Recently, Liu and coworkers proved *in vitro* antiviral potential of HCQ against SARS-CoV-2 infection (Liu et al., 2020c). Currently, there are over 16 ongoing clinical trial researches investigating the potential therapeutic safety and efficiency of CQ for treating COVID-19 spread (Touret and de Lamballerie, 2020). In particular, the administration of CQ and HCQ was shown to block toll-like receptors (TLRs) 7, 8, and 9 mediated signaling responses resulting in impaired host defense mechanisms (Ponticelli and Moroni, 2017); (Schijns and Lavelle, 2020). Moreover, both HCQ and CQ were reported to possess immunomodulatory properties for controlling the cytokine storm i.e., inhibits pro-inflammatory cytokine secretion (IL-6, TNF-γ, and IFN-γ) during viral infection. Specifically, the higher dosage of HCQ and CQ were reported to cause worrisome arrhythmias and even death due to abnormalities in polarization and depolarization of cardiac tissues (White, 2007). Recently, a study on cancer stem cells confirmed mefloquine, an antimalarial compound as a promising drug targeting colorectal cancer stem cells *via* inhibition of RAB5/7 (endolysosomal proteins) (Caly et al., 2020). As the lysosomal-dependent mechanism forms a universal viral infection platform (Pasquier, 2016), hence, examining other autophagy inhibitors may be noteworthy for treating the infectious spread of COVID-19 disease.

## Modulators of the Innate Immune System

### Recombinant Interferons

Type I interferon (also known as antiviral cytokines) is a natural glycoprotein elicited by host cells in the inflammatory response to viral pathogens. Interferon naturally kicks the host immune system into high gear after sensing the presence of any foreign invader. Endogenous human IFNs cytokines such as IFN-α, -β, -ω, -ε and -κ are deeply involved in the innate immunity pathways and acquired responses inducing large signaling proteins which undermines virus replication within the host (Samuel, 2001). IFNs act as potential indispensable antiviral drugs for treating infectious Hepatitis C Virus, SARS-CoV-2 (Cinatl et al., 2003), and MERS-CoV (Sheahan et al., 2020) when utilized alone or in the form of polytherapy. The recent investigation reported potent anticoronavirus activity of exogenous IFN-β against SARS-CoV-2 pneumonia, as IFN-β is efficiently involved in the upregulation of human IFNs subtypes and subsequently augments IFN-mediated innate antiviral cellular response (Scagnolari et al., 2004). Recently, a clinical trial by a UK-based pharmaceutical company (Synairgen) showed that SNG001, multiple sclerosis (MS) IFN-β drug can be potentially used to treat COVID-19 patients. When patients with moderate COVID-19 received SNG001 *via* vapor inhalation, it mitigated critical clinical symptoms in patients accompanied with rapid recovery. Sallard and coworkers reported an acceptable safety profile of IFN-β1 in the preliminary stage of COVID-19 pneumonia (Sallard et al., 2020). Besides, *in vitro* study in human lung tissue (O’Brien et al., 2020) indicated SARS-CoV-2 susceptibility to Type I Interferon than original SARS-CoV (Lokugamage et al., 2020). Specifically, SARS-CoV-2 encodes several Nsps, structural and accessory proteins to target the components of the innate immune system. Encoded Nsps suppress the active expression of IFN-I by inhibiting the activation of STAT transcription factors. In the wake of this evidence, an earlier investigation on novel coronavirus infected ferrets, and lung tissues revealed lower expression of IFN-I, whereas there was a tremendous increase in the production of pro-inflammatory chemokines (Blanco et al., 2015). Another study testing the peripheral blood samples from severe COVID-19 infected patients also indicated impaired IFN-I response (Hadjadj et al., 2020). As an outcome of these investigations, recombinant human IFN-I is actively being trialled as an antiviral approach against COVID-19 pneumonia.

### Natural Killer Cells

Natural killer (NK) cells as key responders of innate immunity induce immunomodulation and rapid viral clearance *in vivo*. Unsurprisingly, elderly and cancer patients with NK cell deficiencies (NKD) are more susceptible to a viral infection with subsequent cases of mortality and morbidity. These NKD constructs a successful route for the propagation of viruses inside the host cell. Therefore, the innovation of the alternative antiviral strategies may have the potential to boost the specific immune system to flatten the drastic COVID-19 curve *via* the implementation of NK cell-based immunotherapies. Currently, due to the paucity of studies, there is a lack of consensus in the context of the functional role of killer cells in the pathophysiology of SARS-CoV-2 infection. A study reported that NK cells and phagocytes trigger the successful elimination of SARS-CoV-2 infected pulmonary cells (Chen et al., 2010). As reported, the innate immune responses were able to reduce SARS-CoV-2 load by increasing the expression of pro-inflammatory cytokines.

Scientists and biotherapy companies have begun designing NK cell-based anticancer medications to treat SARS-CoV-2 infection. Celularity, the USA-based company designed the first cryopreserved placenta haematopoetic stem cell-based NK cell therapy (CYNK-001), an anticancer drug for clinical testing in COVID-19 affected patients (Market et al., 2020).

### Cyclosporin A (CsA)

CsA (immunosuppressant), a well-known calcineurin (CN) inhibitor, functions as a valid antiviral candidate for hepatitis C and MERS-CoV (Santos et al., 2020). In addition, CsA efficiently halts the replication of all CoVs genera, including SARS-CoV at non-cytotoxic concentrations *in vitro* (de Wilde et al., 2018)*;* (de Wilde et al., 2011). While the inhibitory mechanism of CsA is not understood clearly, it is hypothesized that its antiviral property is mediated by targeting cyclophilin-A (Cyp-A) protein pathways as well as inhibition of nuclear factor of activated T cell (NF-AT) pathway. The safety profile of CsA was efficiently evaluated when a series of kidney transplant recipients maintained on CsA medication during COVID-19 treatment showed no evidence of harm (Rodriguez-Cubillo et al., 2020).

### Corticosteroids

Corticosteroids act as adjunctive viral therapy with the ability to provide anti-inflammatory signals by inhibiting the activation of inflammatory mediators secreted during rampant inflammation and infection by the body. They function as a double-edged sword as they are very effective in the treatment of cancer, asthma, auto-immune conditions, and so forth. Lipophilic corticosteroids bind to intracellular corticosteroid receptors (CR) and the complex translocates from the cytosol to the nucleus. Once in the nucleus, the complex binds to the glucocorticoid response element (GRE) and leads to increased gene transcription of several anti-inflammatory cytokines, including IL-4, IL-10, IL-13, and TGF-β (Ramamoorthy and Cidlowski, 2016); (Barnes, 2010). In addition, corticosteroids also increase lipocortin-1 protein synthesis, which in turn curtail the enzymatic activity of phospholipase A2, eicosanoids (local hormones), and platelet-activating factor. Classical multiple mechanisms of corticosteroid action mould them as an effective anti-inflammatory wonder drug at several sites (such as joint, lung tissue, etc).

The SARS-CoV-2 infection causes lung cell disruption, which further triggers local immune response with increased activation of white blood cells (monocytes and macrophages), and cytokine storm syndrome. In most cases, innate and specific immune responses are sufficient to counter the viral pathogens. However, the occurrence of altered immune response is reported with severe development of lung and systemic pathology. As an effective positive anti-inflammatory medication, corticosteroids can be prescribed to ill patients in the early stages of macrophage activation syndrome (MAS), and cytokine storm for reducing immunopathological damage and safeguarding the lungs and lives (Russell et al., 2020). As per other studies preliminary data, the administration of corticosteroid did not show any beneficial effects on lung injury. Consequently, high-dosage leads to serious adverse complications such as mood swings, avascular osteonecrosis, hyperglycaemia, osteoporosis, etc. Although this anti-inflammatory candidate has been reported as a routine treatment of COVID-19 pandemic to combat the extent of inflammation associated with an injury, their use is a point of major controversy, and clinical validation prior to medication administration is highly warranted. Concerning this, 14 clinical research trials have been conducted till date to validate the safety profile and long-term efficacy of the dexamethasone (a cortisol derivative) for the therapeutic management of COVID-19 and heavy death toll. Recently, preliminary positive reports of a randomized, controlled and open-label i.e., recovery trial (NCT04381936, Oxford University) declared dexamethasone drug as the world’s first effective and life-saving candidate to treat critically ill SARS-CoV-2 infected patients (Patel et al., 2020). Around 2,104 patients randomized to receive dexamethasone (6 mg) as a single dose daily for 10 days, demonstrated reduced 28-days mortality risk in SARS-CoV-2 infected patients relying on invasive mechanical ventilation and oxygen. After that, the UK government authorized dexamethasone for the treatment of COVID-19 patients. Unfortunately, no sign of clinical improvement was recorded in moderate and hospitalized COVID-19 patients. The breakthrough discovery of dexamethasone as the first lifesaving drug enlightens the hope to reduce the death toll to a great extent. However, low prices and easy commercialization of this drug will prove as a boon for the lives and economics of the global population (Patel et al., 2020).

### Interleukin (IL)-6 Pathway Inhibitors

IL-6, IL-1, and tumor necrosis factor-alpha (TNF-α) are the three most relevant pro-inflammatory cytokines produced by innate immune cells. In particular, the cytokine storm and increased IL-6 levels are a reliable indicator of worse prognosis among ARDS patients, when compared to mild and non-complicated disease (Voiriot et al., 2017). However, in recent months, maximal levels of pleiotropic cytokine i.e., IL-6 showed a strong negative association with the need for prolonged mechanical ventilation support in patients. The classical IL-6 signaling pathway occurs *via* IL-6 receptors, expressed by monocytes/macrophages, neutrophils, and certainly the other leukocyte populations (Tu et al., 2020). High circulating levels of IL-6 contribute to a faster rate of decline in lung elasticity associated with a higher risk of bronchoalveolar inflammation. Hence, selective therapeutic blockade of IL-6-driven signaling by specific inhibitors may represent a novel and promising strategy to curtail the disease inflicted by inflammation and reduce tissue damage in various organs of ill COVID-19 patients (Rose-John, 2012). In addition, post-IL-6 blockade, a decreased bacterial burden was reported in the lungs of tuberculosis-infected mice.

Tocilizumab (TCZ) biologics is a USFDA approved specific humanized monoclonal antibody for the IL-6 receptor used in the form of the immunosuppressive drug for the management of severe chronic inflammatory diseases (e.g., Rheumatoid arthritis (RA), juvenile idiopathic arthritis (JIA)). IL-6 expression is an important cytokine that induces fever and synthesis of acute-phase proteins, such as ferritin and C-reactive protein (CRP) (Burrage et al., 2020). Exploratory studies revealed heightened cytokine release is positively associated with critically ill SARS-CoV-2 infected patients. TCZ has already emerged as an alternative treatment for the management of COVID-19 patients. In a recent, retrospective case series, 21 severe COVID-19 patients in China received TCZ intravenously (400 mg). Surprisingly, TCZ showed significant clinical improvement, including low oxygen requirement, resolved pulmonary abnormalities, and improved Computerized Tomography (CT) imaging of the chest within a few days (Xu et al., 2020). Another randomized double-blind, controlled trial has been initiated on April 27, 2020, to validate the safety profile and efficacy responses of single-dose TCZ in COVID-19 patients. The TCZ therapy trial is expected to be completed by the end of 2020 and is anticipated to give a positive insight into the clinical utility of TCZ for treating COVID-19 patients.

A B-cell depleting human anti-CD20 mAb, Ocrelizumab has been reported to show an effective therapeutic option for the management of patients suffering from progressive multiple sclerosis (MS) disease and complicated SARS-CoV-2 infection. This wonder mAb therapy reported fast recovery of severely ill COVID-19 patients after 14 days of treatment (Sharun et al., 2020b).

Siltuximab is another most potential USA approved mAb against IL-6 receptor used to treat COVID-19 patients. They are also licensed for the treatment of multicentric Castleman disease. Common toxicities of siltuximab are hypersensitivity reaction and cytopenia (Song et al., 2020). Recently, EUSA Pharma has initiated a study to validate the safety and efficacy of siltuximab in ill SARS-CoV-2 infected patients.

Sarilumab (Kevzara), approved for the rheumatoid arthritis treatment is another IL-6 antagonist. Adverse side effects include a significant reduction in neutrophils, low platelet count, infusion reaction and infection (Song et al., 2020). Sanofi and Regeneron Pharmaceuticals Inc. have conducted a phase 2/3 clinical trial (NCT04315298) of sarilumab in critical COVID-19 patients in the U.S. But this rheumatoid arthritis drug trial did not meet its primary and key secondary efficacy endpoints in SARS-CoV-2 infected patients requiring prolonged invasive mechanical ventilation (Tu et al., 2020).

Another human mAb bevacizumab (anti-vascular endothelial growth factor), underwent a clinical trial study (NCT04275414) in over 20 critically ill SARS-CoV-2 infected patients at Qilu Hospital, Shandong University (China) and proved to be effective in blocking infectivity of lethal SARS-CoV-2 virion (Sharun et al., 2020b). Data on the use of combination therapy of eculizumab and anti-complement C5 successfully recovered ill COVID-19 patients with the reduction in the expression of inflammatory markers along with mean CRP (Diurno et al., 2020). Table 7 outlines the running clinical trials for the evaluation of the safety profile of IL-6 Inhibitory drugs.

**TABLE 7:** Running clinical trial studies for validation of IL-6 Inhibitory drugs (https://clinicaltrials.gov/ct2/results).

Sl. No.	Title	NCT No.	Therapeutic Dose	Phase	Location
1	Evaluating the efficacy of TCZ	NCT04356937	8 mg	3	U.S.
2	Evaluating the efficacy of TCZ and corticosteroids to curtail CoVs infection	NCT04345445	8 mg	3	Malaysia
3	Determination of the efficacy of TCZ Naproxen therapy	NCT04325633	250 mg (BID)	3	Not specified
4	Validating TCZ Baricitinib therapy in SARS-CoV-2 infected patients	NCT04358614	4 mg	2,3	Italy
5	Evaluating safety and efficacy of Baricitinib therapy	NCT04340232	2 mg	2,3	U.S.

## Mitigation of Inflammatory Immune Response

### Mesenchymal Stem Cell Therapy (MSCs)

Stem cell therapy (also known as regenerative medicine) walks in the path of regeneration as well as repair of the cells. Hematopoietic stem cell transplantation is one of the blossomed therapies to date. MSCs are a cluster of clinically utilized multipotent cells used for treating various immune-related complications. MSCs-based immunomodulation therapy exhibits a strong anti-inflammatory response by decreasing the production of inflammatory cytokines and foster tissue repair by producing paracrine factors (Golchin et al., 2020). As per preclinical evidence, MSCs not only play a major role in ameliorating endothelial permeability but also attenuates inflammatory cell infiltration (Jae et al., 2009). Presently, regenerative medicine is also paving its way towards COVID-19 treatment. In the latest investigation, the intravenous infusion of MSCs was proved to be compatible and efficient for fighting against the COVID-19 outbreak. In a recent clinical study conducted by China, seven patients (five patients were critical and two patients were suffering from mild to moderate cases) suffering from COVID-19 pneumonia received an intravenous infusion of MSCs derived from bone marrow. Interestingly, all the patients were cured compared to the placebo control group (Shetty, 2020). This study uncovered the active potential of MSCs in-efficiently retrieving the microenvironment of the lung by recruiting immune cells and treating diseases associated with pulmonary dysfunction (Andriani et al., 2016). Remarkably, MSCs-based therapy as an ideal option gained FDA approval for treating ARDS. At present, ongoing clinical trials are investigating human umbilical cord-derived MSCs and dental pulp-derived MSCs (NCT04293692, NCT04269525, NCT04288102, NCT04302519) (Khoury et al., 2020). Table 8 outlines the running clinical trial studies for validation of Mesenchymal Stem Cell therapy against SARS-CoV-2 virion.

**TABLE 8:** Running clinical trial studies for validation of Mesenchymal Stem Cell therapy (https://clinicaltrials.gov/ct 2/results).

Sl. No.	Title	NCT No.	Therapeutic Dose	Phase	Location
1	Estimating adipose-derived MSCs based therapy	NCT04341610	Dilution of cells in saline (1:1)	1,2	Denmark
2	Estimating efficacy of bone Marrow-Derived MSCs therapy	NCT04346368	1*10E6/kg body weight	1,2	China
3	Estimation of the use of human MSCs	NCT04339660	1*10E6 Uc-MSCs/kg body weight after suspending in saline (100 ml)	1,2	China
4	Evaluation of Uc-MSCs therapy	NCT04333368	1 Million/kg body weight	1,2	France
5	Evaluation of Allogeneic based MSCs safety *via* Clinical trials	NCT04348435	200 million cells as a single dose	2	U.S.

### Passive Immunotherapy Combined With Convalescent Plasma Therapy

Immunotherapy is considered as an effective clinical biology therapy for the treatment of infectious diseases. Immunoglobulins (Igs), also known as antibodies (Abs) are glycoproteins secreted by plasma cells of the adaptive immune system. Abs are primarily categorized into monoclonal Abs (mAbs) and polyclonal Abs (pAbs) and is recommended as a major class of biotherapeutics to neutralize the antigens/pathogens (bacteria and virus), aiding in their destruction. Passive immunization recognizes the antigenic determinants of a foreign antigen and stimulates an immediate immune response against it. Igs can be produced both by natural (isolation from blood) and artificial means (in the lab). Patients who recovered from SARS-CoV infection showed robust neutralizing Abs response against this CoV. mAbs specifically bind to distinct domains of MERS-CoV S protein, including six distinct epitope groups, and interact with the three critical entry functions of the S protein of MERS-CoV: membrane fusion, sialic acid-binding, and receptor binding (Widjaja et al., 2019). Polytherapy of passive immunization combined with poorly as well as with potently neutralizing Abs survived mice from the lethal dose of MERS-CoV infection. Such efficient and target-oriented Abs may enhance humoral immunity against the multiple emerging CoVs pandemics by targeting key S protein epitopes and roles (Dhama et al., 2020a).

**FIGURE 3:**
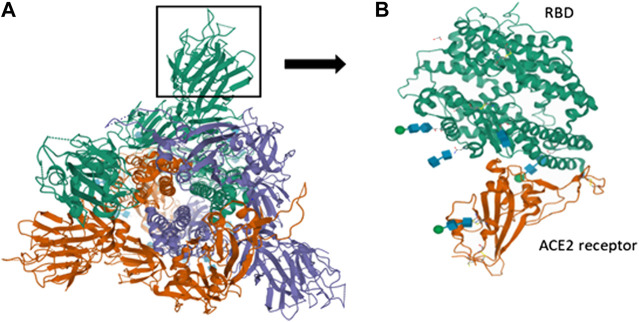
Spike interactions with the ACE2 receptor of the host. (A) Represents the pre-fusion structure of S glycoprotein of SARS-CoV-2 (PDB ID-6VSB). It has a predominant state of trimers with receptor accessible conformation of one of the three RBD due to the upward rotation. The structure represents three chains (A represented in green color, B represented in violet color and C represented in red color); (B) Represents the crystal structure of SARS-CoV-2 spike RBD bound with ACE2 receptor of humans (PDB ID-6MOJ).

The interaction of SARS-CoV-2 with the host receptor (ACE2) marks the beginning of the infection. The spike present on the viral surface (outer) serves as the attachment site and elicits immune response subsequently. The S1 subunit of the S protein contains the RBD which binds to the host receptor. RBD of S protein corresponds to the 74 amino acids involved in the ACE2 receptor binding domain that allows the novel coronavirus to infect human cells. The S2 subunit facilitates membrane fusion and the mode of entry similar to other coronaviruses (Wang et al., 2013). This proves the inherent potential of the S protein in antigenic responses which can be of valuable importance in vaccinology (Peeri et al., 2020). The receptor (ACE2) forms the genomic hallmark explaining the pathogenesis of both SARS-CoV and SARS-CoV-2. As per clinical findings, binding of S protein with cellular ACE2 leads to the negative induction loop hence, ultimately resulting in ACE2 downregulation. This relative downregulation of ACE2 activates angiotensin I (AGI) towards its specific enzyme i.e., ACE, which on the other hand elevates the levels of AGII. Once AGII binds specifically to AGTR1A (receptor), capillary permeability is increased. The structure of the S protein of SARS-CoV-2 is shown in the figure along with the RBD. The protein structure was extracted from PDB (PDB ID-6VSB). The interaction of RBD with the ACE2 receptor of humans is also obtained from the protein database (PDB ID-6MOJ) (Figure 3).

A comparative analysis between RBD of SARS-CoV-2 and SARS-CoV can identify RBD-specific mAbs, and further crossneutralizing SARS-CoV RBD-specific mAbs could be clinically explored to combat symptoms of COVID-19 disease and contribute towards long-term-care protection. Combination therapy of mABs and remdesivir was clinically suggested as a perfect therapeutic option for the treatment of SARS-CoV-2 (Cohen, 2020).

Tian and colleagues reported the potent *in vitro* activity of mAbs (CR3022) in blocking the interaction of S protein RBD with ACE2 (Tian et al., 2020). CR3022 (anti-SARS-CoV antibody) was originally isolated by the Crucell Holland B.V. (Pharmaceutical company, 2006) in Netherland. Another study reported that CR3022 isolated from the COVID-19 patient interacted with the SARS-CoV-2 S protein RBD at a site different from the ACE2 binding site indicating cross-reactivity of the antibody for having similar structural regions on the S proteins of both the CoVs (Yuan et al., 2020). A well-defined interaction interface forms between CR3022 and RBD of SARS-CoV-2 with no interference between ACE2 and SARS-CoV-2 RBD. However, CR3022 binds to RBD, only when RBD is in the “up” conformation. CR3022 Fab specifically binds to SARS-CoV RBD with greater affinity as compared to SARS-CoV-2 due to the presence of non-conserved residues in the epitopes. The potential polytherapy of neutralizing CR3014/CR3022 controls immune escape and extends the protection breadth possibly with a reduced economic cost. Unfortunately, other mAbs directed for SARS-CoV RBD (230, m396, and 80R) didn’t show any positive response against the SARS-CoV-2 pathogen (Santos et al., 2020). In contrast to this pitfall, CR3022 can be recommended as a multifaceted therapeutics against COVID-19. In addition to CR3022, mAbs (F26G18, F26G19, S230) also functions following a similar mechanism. Another potent anti-SARS-CoV S1 pAb T62 that showed a potential inhibitory strategy against SARS-CoV failed to produce a similar lethal effect towards the SARS-CoV-2 virus. Wang and coworkers reported potential neutralizing activity of human mAb 47D11, which specifically targets a conserved epitope on the SARS-CoV-2 S protein. Additionally, TCZ (Actemra) is a recombinant humanized mAbs (IgG1 class) designed for the inhibition of inflammatory protein IL-6, thereby it could dampen the excess secretion of cytokines that may cause long term damage to the lung. As per findings, a threefold increase in IL-6 levels was reported in patients infected with SARS-CoV-2. Currently, various IL-6 inhibitors (tocilizumab, sarilumab, and siltuximab) are under clinical investigation in China for anti-SARS-CoV-2. Regeneron Pharmaceuticals, Inc. recently initiated the first clinical trial of REGN-CoV2 (the cocktail of dual Ab (REGN10933 + REGN10987)) for combating SARS-CoV-2 infection. The placebo-controlled clinical trial included four separate study populations: COVID-19 patients (hospitalized), symptomatic COVID-19 patients (non-hospitalized), asymptomatic subjects (healthcare workers), and asymptomatic individuals in close contact with a COVID-19 patient (housemate). Another Ab, S309 showed potent neutralizing activity towards the SARS-CoV-2 virion by specifically targeting the S protein RBD. In addition, the therapeutic activity of S309 as monotherapy or in combination with S309 containing Ab cocktail, may provide a potential clinical insight for defeating the risk of SARS-CoV-2 virion in high-risk individuals (Pinto et al., 2020).

This therapeutic mechanism works against COVID-19 as these patients suffer a lot from a cytokine storm. The rapid use of versatile mAbs as pharmaceutics during the existing global outbreak will eventually diminish the drawbacks of conventional clinical therapies (lack of specificity, contamination, etc) and may be helpful in the intervention of CoV associated disease.

The administration of potent neutralizing Abs in the form of combination therapy could play an efficient role in reducing CoVs load escaping Ab-dependent neutralization. Even though mAbs have been clinically established as an excellent therapeutic option against COVID-19 in high-risk individuals, the expensiveness, time-consuming production, and the lack of commercially available mAbs either for MERS-CoV and SARS-CoV marks the limitation of a successful therapeutic approach (Shanmugaraj et al., 2020); (AminJafari and Ghasemi, 2020).

NF-kB (nuclear factor kappa-light-chain enhancer of activated B cells) is a protein complex localized in the cytoplasm that plays a key in cytokine production. Therefore, potential inhibition of the NF-kB regulatory pathway may open new therapeutic windows against inflammatory diseases. An *in vivo* study exhibited a promising and long-term protective effect of passive immunization combined with immune serum isolated from MERS-CoV immune camels on MERS-CoV infected mice. However, passive immunotherapy directed administration of convalescent plasma therapy can be another viable and powerful therapeutic strategy to treat critically ill SARS-CoV-2 infected individuals (Zhao et al., 2015); (Zhang and Liu, 2020).

Virion replication and transcription process can be blocked by clinically exploiting Abs/nanobodies which can easily diffuse through the membrane of virus-infected cells and could potently interfere with the pivotal proteins, such as PLpro, 3CL^pro^, and Nsps responsible for the lethal infection (Seesuay et al., 2018). Recovered SARS-CoV-2 infected patients should have a maximal level of pAbs produced by the immune response to curtail new rounds of SARS-CoV-2 infection. Therefore, the administration of convalescent plasma therapy (FDA approved) i.e., successful plasma transfusion from a recovered patient to an infected patient would eventually improve the clinical conditions of virus (SARS-CoV, MERS-CoV, influenza (H5N1, H1N1), Ebola) infected persons with miscellaneous symptoms and inhibit viremia with the overall reduction in the mortality rate. Based on the existing evidence on plasma transfusion therapy, researchers showed the neutralization of novel SARS-CoV-2 (isolated from bronchoalveolar lavage fluid) in an infected patient with early administration of hyper-immune immunoglobulin (convalescent plasma) from immediately recovered patients with simultaneous declination in viral loads (Chen et al., 2020c). Despite the key challenge of plasma collection/fractionation, easy availability of plasma donors with proper clinical conditions, and viral pharmacokinetics, plasma therapy proved to be a great initiative towards the therapeutic world to protect and treat COVID-19 survivors.

In a case study series from China, five critically ill SARS-CoV-2 infected patients under mechanical ventilation received convalescent plasma transfusion with an ELISA IgG titer higher than 1:1000 and neutralizing Abs titer >40 twice on the same day of collection. Out of five, four patients with ARDS recovered, three waened off mechanical ventilation within 2 weeks of treatment and the remaining being healthy (Focosi et al., 2020). Another Chinese pilot study (ChiCTR2000030046) revealed decreased virion load in seven patients associated with improved clinical and radiological results when administered with 200 ml (one dose) convalescent plasma with neutralizing Abs titer higher than 1 : 640. Zhang et al. also reported the recovery of a COVID-19 patient after being administered with convalescent plasma titered with anti-N-protein (Duan et al., 2020); (Zhang et al., 2020a).

Mortality rate to some extent also decreased outside China when two COVID-19 cases with severe ARDS and invasive mechanical ventilation were successfully treated with 250 ml of convalescent plasma titrated with ELISA in South Korea.

As plasma therapy is the most discussed therapy of this era directing towards the fast prevention of coronavirus, Shi et al., reported the identification of two human neutralizing mAbs (termed as CA1 and CB6) isolated from a recovered COVID-19 infectious patient. These two specific mAbs showed potent neutralization activity against SARS-CoV-2 infection *in vitro* (Wrapp et al., 2020). Besides, CB6 demonstrated superior neutralization activity by active inhibition of SARS-CoV-2 infection *in vivo* (rhesus monkey) under prophylactic settings. Structural studies revealed that CB6 efficiently identifies epitopes in novel SARS-CoV-2 RBD that overlaps with cellular ACE2 receptor, and by this means arrests virus/receptor interactions (Wrapp et al., 2020). The problem associated with convalescent plasma therapy is the significant variability of potency in the recovered patient’s sera for antigen neutralization, making it a less viable therapeutic option for treating SARS-CoV-2 infection. Also, it has a limited scope towards combating the global pandemic if the ratio of recovered patients surpasses the infected ones (Saha et al., 2020).

## “Nanosponges” as Decoy Therapy for SARS-CoV-2

Usually, a decoy signifies a thing or a person that intentionally allures something or someone by leading them into a trap (Bradley, 2020). This underlying principle deceives novel SARS-CoV-2 virion under the aegis of decoy receptors (also known as cellular nanosponges). Here, virus-infected host cells are intentionally targeted and designed purposefully to combat the virus (Gershoni, 2008). Cellular nanosponges look like a small polymer-based nanoparticle core efficiently laden within the viral cell membrane displaying receptors similar to the host cell. IL-1R is identified as the first peptide decoy receptors followed by the DcR1, DcR2, and DcR3 of the TNF receptors superfamily (McLachlan, 2020). In general, the infection causing SARS-CoV-2 latches onto the unique proteomic signatures specifically present on the host cell membrane. Once inside the human body, SARS-CoV-2 releases the RNA genome followed by hijacking the host cell. But interestingly here cellular nanosponges deceive SARS-CoV-2 and deviates its path. When the contamination begins inside the human body, the virus interacts with the pseudo receptors (substitute to actual human receptors), and thereby averts further promulgation of the infection. The underlying postulate of this approach shares close proximity with competitive inhibition where decoy receptors fight against the virion by luring it away from cell receptors (Linsky et al. 2020).

To date, cellular nanosponges are of two types i.e., alveolar epithelial type II cells (also known as “Epithelial-NS”) and macrophage nanosponges (also known as “MΦ-NS”). The respective cellular nanosponges were reported to possess invincible antiviral protection to the lungs. In a recent investigation on COVID-19, the PLGA-based nanosponge laden within the membrane of lung epithelial cells were administrated in mice models *via* intratracheal instillation. After three days, a persistent number of blood cells were observed with no evidence of vascular lesion formation or tissue injury. Further, a clinical trial was conducted to evaluate the anti-SARS-CoV-2 potential of epithelial-NS and MΦ-NS. As a result, this report unveiled the equipotency of both cellular nanosponges in SARS-CoV-2 neutralization (Ji et al., 2020).

Natural ACE2 receptors present in the lungs and kidneys not only perform the duty of acting as a gateway for SARS-CoV-2 infection but also regulate blood volume and lowers blood pressure. However, during viral sepsis, these biological activities are altered due to viral interference (Ameratunga et al., 2020). Interestingly, a designed decoy receptor can reinstate these biological activities, leaving the normal ACE2 receptors conducive for their business. Decoy therapy has been approved in compliance with the FDA for several inflammatory and immune-related diseases such as joint pain, inflammation of eyes, recurrent fever, and so forth. This therapy is considered as a novel, promising, and efficient countermeasure to SARS-CoV-2 virus, obstructing COVID-19 spread (Inal, 2020). Recently, a European based Biotech Company; Apeiron Biologics designed an ACE2 decoy for clinical trial study in June 2020 to ensure patient safety with ARDS and pulmonary arterial hypertension *via* intravenous administration. Further, the study demonstrated a tolerable safety profile with minimal known side effects. Another attempt was made by researchers at Rensselaer Polytechnic Institute in July 2020. They reported that heparin, an anticoagulant, bind specifically to SARS-CoV-2 and acts as a promising decoy to outwit the virus. In the wake of this evidence, more clinical trials can be explored based on decoy receptors to efficiently fight against the COVID-19 pandemics. However, the ultimatum task is to ensure targeted delivery without fail.

## HLA Genetics and COVID-19

For meeting the therapeutic demand against coronavirus, the infective host should possess excellent genetic background (as HLA) that triggers antiviral response because impairment of immunological responses further led to an increase in the virus load in the organs (e.g., kidney, intestine) rich in ACE2 expression (Blackwell et al., 2009). The major histocompatibility complex (MHC) class I gene group in humans encodes HLA-A, -B, and -C markers. The HLA molecules are prototypical candidates that help the immune system to recognize the body’s own proteins in contrast to proteins of infectious invaders. They elicit an immune response by binding to the peptides of viral invaders. According to the latest investigations at the University of Geneva (Switzerland), researchers demonstrated HLA variation using bioinformatics analysis to identify those that bind firmly to novel SARS-CoV-2 peptides. Furthermore, the HLA complex was categorized based on how easily they bind to SARS-CoV-2 peptide depending upon the resistivity and susceptibility of the population towards the novel pathogen. As per immunologists, T-cell mediated antiviral responses are associated with different HLA haplotypes with the occurrence of distinct disease susceptibilities. In fact, specific HLA haplotypes have a strong association with susceptibility to influenza, tuberculosis like infectious diseases. The four H1N1 virus infections (HLA-A*11, HLA-B*35, and HLA-DRB1*10) are significantly associated with HLA class I in man, therefore confer increased susceptibility to A(H1N1)pdm09 (Dutta et al., 2018). As a result, there is an urgent need to elucidate the specific HLA loci related to the development of protective immunotherapy against COVID-19 pneumonia. Nguyen et.al demonstrated a comprehensive in-silico analysis showing how HLA variation affects cellular immunological response against peptides of coronavirus. However, the authors predicted HLA B*46:01 with the fewest binding peptides for coronavirus-2, whereas HLA-B*15:03 was identified with a higher capability to present conserved SARS-CoV-2 peptides to immune cells (Nguyen et al., 2020). Hence, HLA typing information would be a feasible helping hand for treating the severity of COVID-19 on a global basis.

### CRISPR Therapy for COVID-19

A novel and powerful genome editing approach as a targeted therapeutic strategy for COVID-19 disease relates to CRISPR/Cas9 technology (Chekani-Azar et al., 2020). Clustered Regularly Interspaced Short Palindromic Repeats (CRISPR) has gained substantial attention due to their efficiency and simplicity. It consists of a guide RNA (gRNA), specific for a DNA or RNA sequence, and a CRISPR associated protein (Cas9). The guide RNA attaches to the complementary target RNA and Cas9 cleaves it, knocking the intended cellular target. The underlying mechanism functions with the degradation of the intercellular viral mRNA (Dara and Talebzadeh, 2020). The β family of coronavirus consists of an ssRNA genome of 26–34 Kb which codes for various structural and Nsps. Naoumov et al, reported the involvement of Cyclophilin (Cyp) protein of the ubiquitous family in the viral replication of SARS-CoV-2. They are a set of peptidyl-propyl-isomerases (PPIases) that functions as chaperones and assists in protein folding, trafficking, and activation of B-cell and T-cell. Cyp inhibitors like CsA, have been observed in various studies by Carbajo-Lozoya et al. (2014). Knockout or knockdown of CypA using CRISPR/Cas9 gene-editing technology adversely affected the critical replication process of α-coronaviruses, feline coronavirus, the arterivirus equine arteritis virus (EAV). Therefore, it was proved that coronavirus replication was directly linked to CypA (Xiang et al., 2020). This inhibitory property was also studied in different cell lines of HCoV-229E and MERS-CoV by Wildjaja et al. (2019). Strong impediment of equine arteritis virus replication and a modest decrease in the growth of MERS-CoV in Huh7-CypAKO cells on the application of CRISPR/Cas9 technology was observed (Nalawansha and Samarasinghe, 2020). The application of CRISPR/Cas13 strategy for diagnosis of COVID-19 disease by antiviral CRISPR in the human cells (PAC-MAN) has been reported by Abott et al. (2020). Two highly conserved regions of the RDRp gene in the open reading frame1 a/b or ORF1 a/b region responsible for viral proliferation and packaging have been recognized that can serve as potential targets for the PAC-MAN approach. In another study, Nguyen et al. (2020) reported that near-about 10,333 guide RNAs have been specifically designed to target ten peptide-coding regions of the SARS-CoV-2 RNA genome. Interestingly, a diagnostic technique based on CRISPR technology called Specific High-sensitivity Enzymatic Reporter unlocking (SHERLOCK), also known as SHERLOCKv2 was reported, which uses multiple Cas13 enzymes and other enzymes, like Csm6, which exhibits RNase activity when activated by some of the Cas13 nuclease products (Lotfi and Rezaei, 2020). The First CRISPR-based detection kit was approved by the FDA that can diagnose the SARS-CoV-2 infection within an hour. Though many studies related to CRISPR stated the innate potential of CRISPR-Cas13, it has unfortunately received less attention at the global level than it should have achieved. However, an effort should be made to develop it as a full-fledged technology (Chen et al., 2020d).

### Adeno-Associated Virus (AAV)-Based COVID-19 Therapy

AAV therapy has been proven to be one of the most successful and established vector gene therapy driven by its safety and efficacy. Adenovirus is a non-enveloped virus that is genetically engineered to deliver DNA to the target cell. Belonging to the parvovirus family, it is known for co-infection with other viruses (Naso et al., 2017). As a current regimen, it has been proven effective against inhibition of the transient receptor potential vanilloid 4 (TRPV4) calcium-permeable ion channel of the SARS-CoV-2 virion. Earlier studies in various preclinical models of lung edema have shown protective medication of virus-mediated vaccine by efficiently targeting the TRPV4 receptor. Hence, the rationale behind AAV therapy is to protect the alveolo-capillary barrier by initiating target mediated treatment of SARS-CoV-2 infection to lessen the pressure on healthcare systems reliable upon invasive ventilator assisted respiration. TRPV4 maintains the integrity of alveolo-capillary barrier along with the regulation of alveolar macrophages and neutrophil granulocytes which on activation leads to barrier disruption by releasing proteases, cytokines, and reactive oxygen species (ROS) (Kuebler et al., 2020). Recently, Phase I clinical trials have been initiated for validating the safety and efficacy of GSK2798745 (TRPV4 inhibitor) in healthy human volunteers and in lung edema patients. The inhibitor can only be administered inside the diseased patient’s body only after the respiratory infection progresses to show SARS-like symptoms (Kronbichler et al., 2020). Strategic application of this therapy can lessen the global burden of deaths which is highly challenging at this point. The development of remedial measures against SARS-CoV-2 infection has focused on antiviral drugs, vaccines, immunomodulatory agents, and protease inhibitors. Targeting TRPV4 for endothelial protection can serve as a promising approach for COVID-19 disease in future (Uppal et al., 2020).

### Toll-Like Receptors (TLRs)

TLRs are located in the endosomal compartment (TLR-3,-7,-8,-9) or on the surface of innate immune cells (TLR-1,-2,-4,-5,-6,-10) such as macrophages and other cells like fibroblast and endothelial cells, are crucial molecules with the inherent potential to recognize pathogen-associated molecular pattern (PAMPs) present within the invading pathogens and elicit an innate immune response with the production of inflammatory cytokines, Type I Interferons and other mediators (Patra et al., 2021). In humans, TLR-3 and TLR-7 are destined to recognize double-stranded RNA (dsRNA) and single-stranded RNA (ssRNA) virus particles respectively suggesting the role of TLR-7 in the clearance of SARS-CoV-2. MyD88-dependent and TRIF-dependent activation of TLR-7 leads to the nuclear translocalization of transcription factors like NF-kappaB, interferon regulatory transcription factor-3 (IRF-3) and -7 (IRF-7) along with the secretion of pro-inflammatory cytokines such as IL-1, -6, TNF-α and type-1 interferons i.e. IFN-α, -β, all responsible for viral clearance. The mechanism to escape the immune system by SARS-CoV suggests a similar mechanism for SARS-CoV-2 also i.e. *via* induced inactivation of TNF-receptor-associated factors (TRAF) -3 and -6, which are key elements for the activation of IRF-3 and -7. This suggests that available TLR-7 agonists such as Imiquimod can potentially serve as anti-SARS-CoV-2 agents (Onofrio et al., 2020).

TLR-4 present on the lungs, and in-macrophages induce strong activation of cytokines leading to elevated secretion of IL-6, 1β, -10, -12, and TNF-α. Studies carried out on IL-6−/− mice suffering from acute respiratory distress syndrome (ARDS) showed that the spread of SARS-CoV was more in IL-6−/− mice as compared to the control (IL-6+ mice). Though the activation of TLR-4 is bacterial dependent, a hypothesis suggests that the activation of TLR-4 in SARS-CoV-2 may be achieved due to the presence of oxidized phospholipids which can trigger TLR-4 activation. SARS-CoV-2 increases the concentration of Neutrophil myeloperoxidase, an enzyme that oxidizes the phospholipids present in alveolar sacks of lungs into oxidized phospholipids leading to strong activation of TLR-4. Hence, TLR-4 possesses the ability to trigger IL-6 production, and TLR-4 agonists like Tocilizumab can be used to target against COVID-19 disease (Felsenstein et al., 2020).

Another study suggests that the immunomodulation of TLR-5 not only prevents the cytokine storm but also activates the immune response against COVID-19 disease. Cytokine storm is commonly reported in SARS-CoV-2 patients, which occurs due to the increased expression of cytokines like IL-6 and rapid recruitment of innate cells like neutrophils due to neutrophil extracellular traps (NETs) and increased concentration of reactive oxygen species (ROS) (Chakraborty et al., 2020). NETs restoration and reduced ROS population can be achieved by Deoxyribonuclease 1, which also helps in TLR-5 modulation. Along with this, the newly developed subunit recombinant vaccine against SARS-CoV-2 contains coronavirus-S1 subunit which is an agonist to TLR-5. A clinical trial of phase II carried out involving 162 volunteers with hepatitis B, proved the efficacy of vesatolimod in combination with other antivirals, with clear signals of increased IFN-stimulated gene mRNA expression with a safety profile. This was also validated for its effect in SARS-CoV-2 patients using combination therapy. Similarly, another drug by the name Eritoran tested in an Influenza mouse model, showed improvement in clinical symptoms, reducing the oxidized phospholipid and cytokine levels and mortality (Biswas and Khan, 2020). Hence, these studies suggest that TLRs-dependent eradication of SARS-CoV-2 can serve as an effective target mediated therapeutic strategy in near future.

## Potential COVID-19 Vaccination Strategies for Vaccine Deployment: A Snapshot of Pandemic Preparedness

**FIGURE 4:**
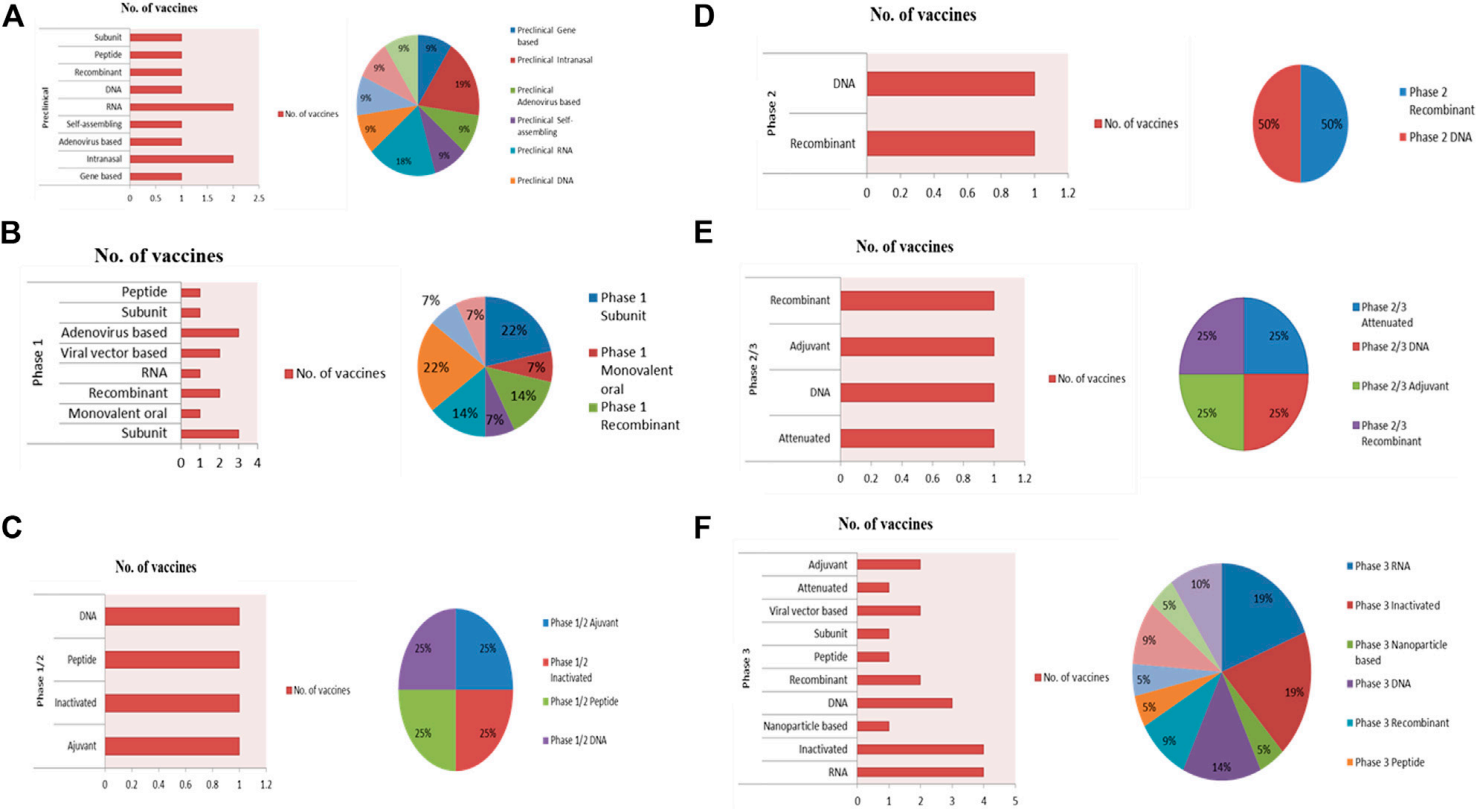
Landscape of COVID-19 vaccine development. As of December 4, 2020, 165 vaccine candidates are undergoing development by academic labs and industry. **(A)**, 86 vaccine candidates have entered pre-clinical trials. **(B)**, 41 vaccine candidates have entered Phase 1 trials. **(C)**, four vaccine candidates have entered Phase 1/2 trials. **(D)**, 17 vaccine candidates have entered Phase 2 trials. **(E)**, four vaccine candidates have entered Phase 2/3 trials. **(F)**, 13 vaccine candidates have entered Phase 3 trials. The vaccine candidate data was compiled from searching vaccine tracker resources: Regulatory Affairs Professionals Society (https://www.raps.org/news-and-articles/news-articles/2020/3/covid-19-vaccine-tracker), BioCentury (https://www.biocentury.com/clinical-vaccines-and-therapies), and World Health Organization (https://www.who.int/emergencies/diseases/novel-coronavirus-2019/covid-19-vaccines).

Considerable global efforts have been undertaken by the researchers around the clock to hasten the development and manufacture of a much-needed vaccine against COVID-19, to obviate the serious risk of a pandemic and most of the developing vaccine candidates have been using the SARS-CoV-2 S protein as the major target (Dhama et al., 2020c). Vaccine developments necessitate the recognition of an immunogenic and establishment of a full-fledged vaccine after assessments through different studies to regulate the safety and efficacy of the resultant vaccine (Rappuoli, 2001). Blossoming of a novel vaccine demands its approval in the following stages: Exploratory stage, Pre-clinical stage, and clinical (Phase I, Phase II, and Phase III) stage (Thanh Le et al., 2020) (Figure 4). The field of vaccinology is yet to set its foot on the zenith of success. As of November 27, 2020, the worldwide COVID-19 vaccine landscape includes 55 vaccine candidates which are in the human clinical trials and 87 are in the preclinical stage of their development process. Currently, Moderna’s mRNA-1273, Ad5-nCoV (CanSinoBIO), ChAdOx1 (University of Oxford), AstraZeneca’s AZD1222, and Pfizer and BioNTech's BNT162 have entered phase III human clinical trials. The candidate COVID-19 vaccines which are in the pipeline are specifically based upon live attenuated viruses, DNA, RNA, nanoparticles, replicating and non-replicating viral vectors, immunogenic adjuvants (Dynavax, Novartis, GSK), protein sub-unit and so forth, each displaying their key benefits and hindrances before its launch in the current global market (Table 9) (Liu et al., 2020a); (Bhandari et al., 2021). The intricacies of the elaborated procedures have confined the evolution of vaccines. At present, there is no innovative vaccine with full proven potency for SARS-CoV-2. The rational design of potential vaccine-mediated protection works on the principle of vital immune generation *via *the generation of desired persistent B-cell and T-cell mediated immune responses. Traditional vaccines bridge the gap at the cost of time, finance, and safety. Therefore, the pressing priority is to design a novel vaccine that is adapted to sow the seeds of immunity against the emerging pandemic (Wu, 2020). The immune-informatics based approach has paved the way for the SARS-CoV-2 epitope identification for vaccine candidate production, which can further be used for the identification of significant viral T and B-cell epitopes (Baruah and Bose, 2020).

**TABLE 9:** Outlook of COVID-19 vaccine development.

Vaccine Candidate	Country	Manufacture (Sponsor)	Phase
Sputnik-V/Gam-COVID-Vac	Russia	Gamaleya research Institute	3
Inactivated vaccine (COVID-19 vaccine)	China	Wuhan Institute of biological products/Sinopharm	3
ChAdOX1-S	U.S.	University of Oxford/AstraZeneca	3
Adenobased (rAd26 + rAd5-s)	Russia	Gamaleya research Institute	3
Ad26CoVS1	Belgium	Janssen pharmaceutical companies	3
Full length recombinant SARS-CoV-2 glycoprotein nanoparticle vaccine adjuvanted with Matrix M	UK	Novavax	3
LNP-encapsulated mRNA	USA	Moderna/N1 AID	3
Adjuvanted recombinant protein (RBD-dimer)	China	Anhui Zhifei longcom biopharmaceutical Chinese Academy	2
mRNA vaccine	Germany	Curevac	2
DNA-vaccine (GX-19)	South Korea	Genexine-consortium	½

## Contemporary Vaccines

Traditionally, the generation of active immunization against viral illness using an attenuated form of whole pathogens *via* physical or chemical strategies has resulted in approved clinical treatments. Further, many repurposed mammalian based viral vectors (such as oncolytic herpes simplex virus) have been approved for clinical treatments. Contemporary vaccines share an advantageous position, and in this comprehensive review, we focused on the attempts of the development of live-attenuated, inactivated, and viral vector-based vaccines in shaping the reality of the COVID-19 vaccine.

### Inactivated Vaccines

Inactivated vaccines (IVs) are heat or chemically (formaldehyde or β-propiolactone) treated live virus particles as a means to reduce virulence. The formulation of these vaccine candidates is incapable to undergo an active replication process and considered to be safe than live-attenuated vaccines (LAVs), but their inactivation is associated with a lower immunoreactivity. However, this pitfall requires careful planning of multi-dosage regimens to develop long-term active and protective immunological memory; also, there is a critical need for novel immunologic adjuvants in vaccine formulations to improve the efficacy of immunization in the elderly population as a result of immunosenescence as per the reports of Phase 1 (NCT04352608) and Phase 2 (NCT04383574) trials (Ciabattini et al., 2018).

### PiCoVacc

Recently, Sinovac Biotech Ltd. (NasdaqGS:SVA), a private China-based biopharmaceutical company, developed a formalin-inactivated and alum-adjuvanted vaccine candidate pertinent only in immunosuppressed patients. The purified inactivated vaccine was designed on a pilot-scale against SARS-CoV-2 infection under the tradename “CoronaVac (formerly named as PiCoVacc). Its immunization induces the production of specific neutralizing antibodies response in rats, mice, and non-human primates (Yang and Wang, 2020). Partial or complete protection against SARS-CoV-2 infection was achieved in macaques with the administration of two different doses (3–6 µg per dose) in three subsequent phases in clinical assessments. Currently, Phase-3 trial (NCT04456595) of Sinovac’s COVID-19 vaccine is underway in collaboration with Instituto Butantan in Brazil followed by others in Indonesia (NCT04508075) and in Turkey (NCT04582344).

### Live-Attenuated Vaccines

Attenuated vaccines (or LAVs) are viable and reproducing, but avirulent viruses are developed usually by repeated culturing with human or animal cells. They are more antigenic than IVs therefore, generate a vigorous immune response (Dhama et al., 2020c). The Chinese Centers for Disease Control and Prevention, Wuhan Institute of Virology, Chinese Academy of Sciences, Zhejiang University, Codagenix Inc., and Serum Institute of India, Ltd. are in the process of developing attenuated vaccines (Shereen et al., 2020). Immunodominant epitopes of N protein and E protein are potential targets for designing novel attenuated vaccines against SARS-CoV-2 infection. A reverse genetics approach was used to counteract the exonuclease effects of Nsp14. Another strategy aimed at deleting the E protein of SARS-CoV-2. The design of LAV aims for single-dose immunization without illness. The emergence of promising LAV as the most mature technology establishes it as one of the frontrunners in vaccine development to mitigate the ongoing SARS-CoV-2 infection. Furthermore, LAVs technology requires cold chain management and logistics. The loss of potential effectiveness and reproductive capacity of progeny virion particles imposes serious regulatory challenges during the development of new vaccines. Latest genetic code expansion technology is being commonly applied to generate genetically stable and highly reproductive LAVs. Recently, a genomics-based approach has enabled the engineering of synthetic recombinant SARS-CoV-2 virion from viral DNA fragments (Thi Nhu Thao et al., 2020). Employment of these novel strategies is the need of the hour for the rapid generation of SARS-CoV-2 LAVs.

### DelNS1-SARS-CoV-2-RBD

This live-attenuated influenza virus-based vaccine candidate contains carboxy-terminal deletions in the NS1 protein which expresses the RBD domain in its outer surface and is further cultivated *in vitro* in Madin Darby Canine Kidney Cells (MDCK) cells. Live-attenuated influenza vaccine is significantly more immunogenic and safe as compared to wild-type and can be effectively administered in the form of an aqueous intranasal spray (Zhu et al., 2020a).

### Viral Vector-Based Vaccines

Viral vector-based vaccine candidate intends to be a promising prophylactic and therapeutic solutions against the ongoing COVID-19 pandemic. They specifically deliver and express the therapeutic genes to target cells/organs followed by comprehensive induction of immune responses. They allow a high-level expression of antigenic protein and serves as another major weapon against SARS-CoV-2 infection as these vaccines trigger and prime the cytotoxic T cell (CTL) responses which ultimately destroy and eliminate the persistent virion infected cells (Zhu et al., 2020a). Several replication-incompetent adenoviral vectors are in development for COVID-19 vaccines. Adenovirus-based vectors offer significant advantages such as inherent immunostimulatory activity, broad tropism, and scalable manufacturing. Challenges associated with using the adenoviral vector platforms include pre-immune humans, which may dampen the overall adenoviral vector efficacy.

### Ad5-nCoV

China’s CanSino Biologics developed a recombinant, replication-incompetent adenovirus type-5 vector (Ad5)-based vaccine named Ad5-nCoV in collaboration with the Institute of Biology at the country’s Academy of Military Medical Sciences (AMMS) expressing the recombinant S protein of the SARS-CoV-2 virion. Efficient preparation of this vaccine involves cloning of the appropriate full-length S protein gene with plasminogen activator signal peptide gene in the E1 and E3 genes-deleted Ad5 vector. This vaccine was designed using the Admax system (MicrobixBiosystem Inc.) (Zhu et al., 2020a). In May, data from a Phase 1 safety trial resulted in a promising 4-fold increase in the S protein and RBD-specific neutralizing Abs response within 14 days of immunization. Furthermore, CD4^+^ cells and CD8^+^ cell responses enhanced around day 14 after vaccination (Zhu et al., 2020a). In an unprecedented move, on June 25 Ad5-nCoV gets a Chinese military nod to successfully utilize it for a year as a “specially needed drug”. Starting in August, CanSino Phase 3 COVID-19 vaccine trial in about 40,000 participants began in Saudi Arabia, Russia, and Pakistan (NCT04526990).

### Coroflu

Coroflu, a unique nasal vaccine against ongoing COVID-19 pandemics was developed and tested by an international collaboration of virologists at the University of Wisconsin-Madison in collaboration with vaccine makers (FluGen, Bharat Biotech). It was built on a flu vaccine “backbone” called as M2SR, which is a self-limiting form of the flu virus designed by insertion of the SARS-CoV-2 S protein gene sequence. Coroflu expresses major influenza virus antigen i.e., hemagglutinin protein, thus confers immune response against both coronavirus as well as influenza. MSR2 lacks the M2 gene, thereby inhibiting the replication potential of the virus. Intranasal administration of the vaccine candidate mimics the natural route of viral infection, thereby activating the multimodal immune system with a higher immunoreactivity compared to the intramuscular shots (UW–Madison 2020, FluGen, Bharat Biotech to develop CoroFlu, a coronavirus vaccine).

### LV-SMENP-DC

This vaccine is made by Shenzhen Geno-Immune Medical Institute by modifying the Dendritic cells (DC) with the lentiviral-based vector expressing the conserved domain architectures of the SARS-CoV-2 structural proteins and protease (SMENP) minigenes accompanied by strong immune-modulatory genes. Antigens are expressed on the surface of the antigen-presenting cells (APCs) on subcutaneous inoculation of the vaccine, which ultimately elicits an immune response by activating CTL (Thanh Le et al., 2020).

### ChAdOx1

AstraZeneca in collaboration with Oxford University developed ADZ1222 (previously known as ChAdOx1) COVID-19 vaccine candidate, a Chimpanzee adenovirus-based vaccine using codon-optimized SARS-CoV-2 structural S glycoprotein antigen and synthesized with the tissue plasminogen activator leader sequence (TPA) at the 5’ end. S protein gene of SARS-CoV-2 is inserted into the E1 locus of ChAdOx1 adenovirus genome for the construction of adenovirus vector genome in the Bacterial Artificial Chromosome. Viral reproduction was further allowed in T-Rex 293 HEK (Human Embryonic Kidney 293) cell lines followed by purification with caesium chloride (CsCl) density gradient ultracentrifugation. Intra-muscular vaccination during preclinical trials showed the absence of sub-genomic RNA (sgRNA) resulting in escalated immunogenicity against the infectious virion. The titers of neutralizing antibody provided partial protection in rhesus monkeys in the 5–40 range. A moderate hike in the respiratory rates in three out of six macaques with no change in nasal viral RNA quantity was recorded for vaccinated and control groups. ADZ1222 vaccine cleared the Phase 1/2 clinical trials successfully after eliciting the production of pseudovirus-neutralizing and live virus-neutralizing Abs after the 28 days of post-vaccination. But later on, the trial procedures halted for a few months due to the development of neurological complications and severe infections in a COVID-19 individual . The United States of America FDA approved Phase 3 clinical trials of the ADZ1222 vaccine (Wang et al., 2020b). This vaccine also has a very high affinity for gastrointestinal and respiratory sites that expresses the ACE2 receptor. This characteristic makes it fit for use as a good vaccine candidate (Cao et al., 2018). In India, this vaccine candidate is being developed jointly by AstraZeneca and the Serum Institute of India and goes by the name Covishield. A phase 3 clinical trial (NCT04516746) enrolling more than 40,000 patients are underway. Phase 3 study trials which were suspended in the United States and some study sites in India have now resumed. On Dec 7, the Serum Institute of India announced emergency use authorization of the ChAdOx1 vaccine from the DCGA of India. In Australia, the Therapeutics Goods Administration (TGA) has granted a provisional determination to AZD1222.

### Subunit Vaccines

Subunit vaccines are the one which constitutes synthetic peptides, recombinant antigenic proteins, or DNA/RNA fragments necessary to prime invigorating long-lasting therapeutic immune responses in the immune-compromised patients when administered with molecular adjuvants to potentiate the vaccine-induced immunoreactivity (Wang et al., 2020b). The addition of adjuvants to vaccines ameliorates the immunomodulatory cytokine response as well as helps in overcoming plausible flaws and potential weaknesses of the protein-based subunit vaccines (Cao et al., 2018). Several licensed adjuvant vaccines under the trade name AS03, MF59, and CpG 1018 are in the process of development by GlaxoSmith Kline, Seqirus, and Dynavax . SARS-CoV-2 S protein forms the suitable antigen for inducing specific therapeutic antibodies against NTD, RBD, and fusion peptide (FP) which can neutralize SARS-CoV-2. The virion mediates its entry through endocytosis into the host cells using the S protein-mediated binding to the hACE2 receptor. Therefore, the full length S protein and its antigenic components serve as the promising targets of the subunit vaccines (Wang et al., 2020b). The dynamic S protein possesses 2 conformational states, including pre-fusion and post-fusion conformations. Therefore, it is of utmost importance for the antigen to maintain its surface chemistry and profile of the original pre-fusion conformation of the S glycoprotein to conserve the highly efficient epitopes to elicit good quality and quantity of antibody responses. Targeting the masked receptor binding motifs as an antigen will improve the ignition of the neutralizing antibody response and enhance the overall protective efficacy, effectiveness, and safety of the vaccine candidate.

### NVX-CoV2373

In March, Maryland-based Novavax announced the development of a stable, pre-fusion S protein nanoparticle-based immunogenic vaccine candidate named NVX-CoV2373 (Emergent BioSolutions) against the ongoing COVID-19 pandemics (Coleman et al., 2014). Stable expression of the protein resulted in the Baculovirus Expression Vector System (BEVS) (Tu et al., 2020). Furthermore, Novavax’s patented saponin-based adjuvant (Matrix-M) showed potent immunogenicity against the SARS-CoV-2 S protein via the elicitation of potentially high levels of neutralizing antibody response. In May, the company launched COVID-19 vaccine trials followed by the investment of $384 million by the Coalition for Epidemic Preparedness Innovations in the vaccine. In July, Novavax was awarded $1.6 billion by the U.S. government towards the clinical trial studies and manufacturing of the NVX-CoV2373 vaccine candidate. In August, Novavax launched a Phase 2 clinical trial in South Africa after obtaining promising results from preclinical studies in monkeys and humans. In the following month, the company officially began a Phase 3 trial in the United Kingdom enrolling up to 15,000 participants. Another larger Phase 3 trial is expected to launch in the U.S. by the end of December. In September, the company reached an agreement with the Serum Institute of India, to enable them to produce as many as 2 billion doses a year. If the trials succeed, Novavax expects to deliver 100 million doses for use by the first quarter of 2021 in the U.S. On November 4 another agreement was announced to deliver 40 million doses to Australia.

### Molecular Clamp Stabilized S Protein Vaccine Candidate

This subunit vaccine candidate was developed by the University of Queensland in collaboration with the GSK and Dynavax which would maintain the pre-fusion conformation of the S protein in a stabilized form. The use of vaccine adjuvant platform technology (AS03 Adjuvant system) can further strengthen the response of the vaccine candidate as well as mitigate the amount of vaccine/dose required (Lee et al., 2020). The development of a stabilized pre-fusion, recombinant viral protein sub-unit vaccine candidate based on the Molecular Clamp technology is underway by the University, However, this unique technology proved its efficacy in the production of high antibody response for the neutralization of SARS-CoV-2 virion (Tu et al., 2020).

### PittCoVacc

This recombinant SARS-CoV-2 vaccine (University of Pittsburgh) involves the administration of rSARS-CoV-2 S1 and rSARS-CoV-2-S1fRS09 (recombinant immunogens) utilizing Micro-Needle Array (MNA). Immunization of mice with the vaccine through a fingertip patch resulted in the higher production of the antigen-specific Abs followed by the statistically significant improvement in the pre-clinical trials at the end of 2 weeks. Furthermore, the vaccine retained its high-level immunogenicity even after γ-radiation exposure. Also, it was observed that sterilization using gamma radiation maintained the immunogenicity of the vaccine. The significantly higher antibody titers at the early stages of infection support MNA-SARS-CoV-2 vaccine feasibility (Chary et al., 2020).

### Triple Antigen Vaccine

The vaccine developed by Premas Biotech, India is a multi-antigenic virus-like particles (VLPs) vaccine prototype with a recombinant co-expression of recombinant S, M, and E protein of SARS-CoV-2 in an engineered *Saccharomyces cerevisiae* expression platform (D-Crypt™). Biophysical characterization of VLPs using Transmission Electron Microscopy (TEM) supported the entry of the prototype into the pre-clinical trials as a potential vaccine candidate with a safety profile accompanied with feasible and cost-effective manufacturing on a large scale (Arora et al. 2020).

## New Generation Vaccines

The nanoscale viruses can be regarded as potential natural occurring nanoparticles (NPs) and the LAVs, IVs, and viral-based vectors are termed as efficiently engineered nanotechnologies. The virus particles and the NPs function at the same nanoscale, this property drives powerful implementation of versatile nanotechnology-based vaccine development and immuno-engineering through a bottom-up approach. NPs (natural or synthetic) mimic the natural structure of viruses whereas nano-chemistry, chemical biology and biotechnology enable the productive engineering of “next generation” vaccine candidates to combat global killers. Formulation of a new generation vaccine targeted against SARS-CoV-2 is completely based on the homology studies of SARS-CoV and MERS-CoV.

### mRNA Vaccines

Non-infectious mRNA technology emerges as a potential non-integrating platform with negligible risk factors for infection or insertional mutagenesis. mRNA vaccine technologies serve as a new era in vaccinology which works in the direction of production of artificial mRNA of the deadly virus that codes for antigenic proteins. The synthetically produced mRNA when administered into the host produces the same viral proteins and elicits effective cell-mediated and humoral-mediated adaptive immune responses. Complete genome sequencing of the virus fast-tracked the design of such type of novel vaccines (Liu et al., 2020a). mRNA immunogenicity can be down-modulated and altered to enhance vaccine stability. The anti-vector immunity of the mRNA is avoided due to its intrinsically safe and minimal immunogenic genetic vector, therefore, allowing repetitive administration of the mRNA vaccines (Pardi et al., 2018). The field of mRNA vaccine has experienced rapid development because of their flexibility to mimic the antigen structure and expression relatively in the course of natural infection, speed (Mulligan et al., 2020). However, a unique advantage of these nucleic acid vaccines (mRNA and DNA) is that in addition to antibody and CD4^+^ T cell responses, these vaccines also aim at eliciting robust CD8^+^ T cell responses, essential for virion eradication. Recently, in the U.S, Moderna’s mRNA-1273 designed by Moderna Therapeutics, funded by Coalition for Epidemic Preparedness Innovations (CEPI) underwent its Phase 1 clinical trial on March 16, 2020, as an advanced candidate and was successfully approved by the National Institute of Allergy and Infectious Diseases (NIAID), part of the National Institutes of Health (NIH) for further clinical assessments.

### mRNA-1273

Boston-based Moderna’s mRNA-1273 vaccine candidate is a synthetic mRNA coding full-length, the stable pre-fusion conformation of the SARS-CoV-2 S protein encapsulated within a lipid nanoparticle that is considered to be safe for human use (Yadav et al., 2020). This vaccine elicits a highly potent antiviral response against S protein. Furthermore, this vaccine maintains its safety profile because it is not engineered using the inactivated pathogens or the sub-units of the live pathogen. In collaboration with the National Institutes of Health, Moderna yielded promising results of mRNA-1273 in monkeys suffering from the SARS-CoV-2 infection. A Phase 3 clinical trial (NCT04470427) of this vaccine began on highly infected 30,000 SARS-CoV-2 patients on July 27. On November 16, in an interim analysis of the Phase 3 COVE mRNA-1237 met its primary endpoint with an efficacy rate of 94.5%. After three months of the vaccine administration, the company found that the patients were able to produce a strong immune response against the virion. Moderna announced a vaccine trial on December 2 on adolescents (12–18 years). The company reached an agreement with the European Commission to supply 160 million doses on Nov 25. It also has similar deals with Japan, Canada, and Qatar. Immunization of this vaccine in mouse models prevented the replication process of the virus in the lungs. Graham and coworkers showed that mRNA-1273 produces potent neutralizing antibody response including CD8^+^ T cell responses against infection of SARS-CoV-2 in the mice lungs and nose (Corbett et al., 2020).

### BNT162b2

On November 9, the New York-based Pfizer and the German company BioNTech developed a vaccine candidate named as BNT162b2 which is a codon-optimized mRNA-based vaccine encoding for the critical target i.e., full length S protein of SARS-CoV-2. BNT162b2 provokes immune response against SARS-CoV-2 RBD with the involvement of T4 fibritin (foldon)trimerization domain. The intramuscular vaccine has been licensed in China by Fosun Pharma. Efficient encapsulation of mRNA vaccine candidates inside the ionizable cationic lipid NPs (80 nm) improves its effective delivery. The Phase 1/2 clinical trials reported increased elevation of RBD-specific IgG antibodies with geometric mean titer high as 8 to 46.3 times titer of convalescent serum, whereas the geometric mean concentration (GMC) of the SARS-CoV-2 neutralizing antibodies were reported to be 1.8 to 2.8 times the convalescent serum panel (Mulligan et al., 2020). The launch of Phase 2/3 trials (NCT04368728) on 30,000 participants was announced by the companies on July 27 in the U.S., including countries like Germany, Brazil, and Argentina. On December 8, the FDA determined the overall efficacy of the vaccine to be 95% after the release of interim results from the Phase 3 trials. On December 8, William Shakespeare, age 81, was first immunized with the vaccine; shortly afterward Bahrain approved the emergency use of the vaccine on 4 December. On December 9, Canada too granted emergency authorization of the vaccine candidate followed by the shots administration. The companies predict to globally supply over 1.3 billion vaccine doses by the end of 2021.

#### DNA vaccines

DNA vaccine, the most revolutionary vaccination approach consists of genes encoding for the required antigenic proteins that are linked to their promoters and inserted directly in the cells of the host with the help of a gene gun for elicitation of the adaptive immune response. The immune response generated by this method lasts for a longer period (Ahn et al., 2020). Furthermore, the antigenic material undergoes the process of endocytosis by the immature DCs, hence presenting the antigenic material to the MHC2 and MHC1 antigens expressed on the surface CD4^+^ and CD8^+^ T cells, respectively which further leads to the stimulation of active adaptive immune responses (Hobernik and Bros, 2018). One such nucleic acid-based vaccine designed for SARS-CoV-2 was endorsed as “INO-4800” by Inovio Pharmaceuticals. It is cost-effective, highly stable at room temperature, storage resistant, and is easily purified. Several factors should be taken into consideration like the route of administration, the amount of plasma delivered, and the adequate interval of time during vaccine administration. However, it is much more beneficial as compared to conventional vaccines (Chary et al., 2020).

### INO-4800

It is a prophylactic DNA-based vaccine developed by Inovio Pharmaceuticals to combat the ongoing COVID-19 pandemics with the utilization of the codon-optimized S protein sequence of the SARS-CoV-2 with a leader sequence of IgE (Tebas et al., 2020). The IgE-S protein sequence was subjected to restriction digestion using BamHI and XhoI. The digested DNA segment was inserted into expression vector pGX0001 under the governance of IE CMV, and BGH polyadenylation signal. Preclinical trials report the production of functional antibodies and T cell response within 7 days after the vaccination Phase 2/3 trial (Smith et al., 2020). Phase 1 clinical trial was CEPI funded, which involved 40 adults who received two-dose shorts of 1–2 mg within 4 weeks *via* intradermal injections by INOVIO's CELLECTRA^®^ 2000. It successfully had a safety profile and well tolerability while the Phase 2 trial is still underprocess. The approval of this vaccine was put on partial hold due to issues regarding its delivery device on September 28. Later on, it was approved by F.D.A. on November 16 to begin phase 2/3 trial (Tebas et al., 2020).

### Peptide Vaccines

The safety of the vaccine stands out as an important factor during the span of vaccine design. Vaccine construction to represent the whole structural motifs of S protein eliciting the production of broad-spectrum antibodies and cellular responses have indicated a higher risk of antibody-dependent enhancement (ADE) in the case of SARS-CoV and MERS-CoV vaccine (Wang et al., 2016); (Chen et al., 2017). The former led to increased incidences due to the production of non-neutralizing antibodies whereas the latter caused life-threatening allergic inflammations. As such there is no full-proof evidence; however, data collected from immunological patients reflects co-relation between high IgG levels and worsening of the disease outcome. Therefore, the development of a peptide vaccine against SARS-CoV-2 S protein might serve as a safe alternative (Lucchese, 2020); (Baruah and Bose, 2020). There are various epitopes of S protein that are computationally predicted using in-silico approaches. Experimentally configured peptide epitopes obtained from COVID-19 patients by screening for neutralizing antibodies proved the existence of useful epitope regions. Recently, the National Institutes of Health (NIH) funded the La Jolla Institute for Immunology (LII) in this regard. Peptide-based vaccines share an advantageous position due to ease of vaccine design, faster validation, and rapid manufacturing. These vaccine formulations can be obtained as peptide plus adjuvant mixtures or peptides that can be administered by a specific nanocarrier or be encoded by a nucleic acid. Several peptide-based vaccines as well as peptide-nanoparticle conjugates are in the process of clinical testing and development for targeting cancer and several chronic diseases (Bezu et al., 2018).

## Plant Vaccines

Plant vaccines are prepared by the integration of disease-specific antigenic genes into the genome of the plant by different methods. They have high therapeutic applications for the treatment of various diseases (Daniell et al., 2009). Recently, a recombinant plant vaccine with intermodal mechanisms eliciting B-cell and T-cell mediated immune response has been designed by Medicago containing virus-like particles (CoVLP) with an adjuvant system of GlaxoSmithKline that underwent phase 1 trials and later will be tested in three doses (3.75, 7.5, and 15 μg) in healthy individuals with age range 18–55 years (Rosales-Mendoza et al., 2020). Cytotoxicity of various plant compounds like Ferruginol, 8β-hydroxyabieta-9, 13-dien-12-one, 7β-hydroxy-deoxy-crypto japonal, 3β,12-diacetoxyavieta-6,8,11,13-tetraene, betulonic acid, and savinin in inhibiting SARS-CoV replication in the kidney Vero E6 cells of African green monkey has been observed by Wen *et al.in* 2007 (Tahir ul Qamar et al., 2020). Also, betulonic acid and savinin were responsible for restricting 3CL^Pro^ activity with EC_50_ of 10 and 25 µm. Wen et al. (2011) reported that ethanolic rhizome extract of *Cibotium barometz* and methanolic tuber extract of *Discorea batatus* played an important role in preventing viral growth. Essential oil by the name Laurus nobilis showed inhibition of SARS-CoV cells with an EC_50_value of 120 μg/ml and an SI value of 4.16. Essential oils derived from many medicinal plants like Citrus spp, *Hyssopus officinalis*, Illicium spp, mayweeds, tea trees, Mentha spp, Santalum spp, Pinus spp, thymus, and ginger are documented with antiviral activities which can be tested against SARS-CoV-2 (Ngwa et al., 2020). Disruption of the lipophilic phospholipid bilayer of SARS-CoV by monoterpenes, oxygenated sesquiterpenes, and phenylpropanoids of essential oils disrupt the structure of the viral envelop which interferes in viral entry. Inhibitory effect of M^pro^ activity of SARS-CoV-2 has been reported by Tripathi et al. (2020) due to the action of a bioactive compound Withanoside V in Ashwagandha which can combat the novel coronavirus (Chikhale et al., 2020). Asymptomatic symptoms of COVID-19 can be relieved by the use of Sanjeevanivati, Chitrakadivati, and a combinatory treatment of Guduchi (Tinospora cordifolia) and Shunthi (Zingiber officinale) and Haridra (Curcuma longa) for suppressing viral growth. Certain promising phytochemicals such as scutellarein, silvestrol, tryptanthrin, saikosaponin B2, quercetin, myricetin, caffeic acid, psoralidin, isobavachalcone, and lectins (griffithsin) show the effective viral inhibitory effect to combat CoVs (Jahan and Onay, 2020).

## Adjuvanted Vaccines

Adjuvanted vaccines fall under the category of fusion vaccine. It involves drug delivery with immunomodulatory function in combination with the antigen. A very low concentration of the antigen is enough to generate ample immune response. Several potential adjuvants have been identified for designing a vaccine against SARS-CoV-2 infection (Zhang et al., 2020b). Some of the common vaccine adjuvants are alum, MF59^TM^, Montanide ISA51, Deltalin, TLR3, Protollin, CoVaccine HT^TM^. A report by Wang *et al.* suggests that ovalbumin (adjuvant) was used as a model subunit antigen to deliver aluminium nanoparticles which induced both Th1 and Th2 hypersensitive immune response over conventional alum which is only capable of eliciting Th2 response.

## Nanoparticle-Based Vaccines

Nanoparticle-based vaccines have immense potential to serve as a vaccine candidate against any infectious disease. The nanoparticle encapsulates an antigen accompanied by the stabilizing agent that gets released into the body of the vaccinated host after its delivery. It protects the premature destruction of the antigen before it reaches the target points (Abd Ellah et al., 2020). They are of several types such as polymers, metals, lipids, and proteins. They mimic the actual virus and stimulate the antigen-specific proliferation of lymphocytes. When delivered orally, they also generate systemic responses against the virus (Itani et al., 2020). First, the S protein’s pre-fusion structure was expressed in mice.

In the next step, they were immunized with the lipid-based nanoparticle-containing ssRNA with doses ranging from 0.01 to 10 μg. After 6 weeks it was observed that the mouse generated large quantities of IgG antibodies specific for SARS-CoV-2. Also, the amount of antibody produced by the vaccinated mouse and recovered COVID-19 patient were strongly correlated in terms of producing neutralizing antibodies. This vaccine also underwent several testing for SARS-CoV, HCoV-229E, and MERS-CoV and was effective to a considerable extent. The vaccinated mouse was recorded to have a high concentration of cytokines (IFN-γ) as stated by the ELISpot quantification report. Also, high levels of IL-6, MIP- 1β, RANTEs, IFN-β, and IP-10 were found in the blood serum of the mouse. High-dose dependent delivery induced large titers (>10^−6^ng ml^−1^) of neutralizing antibodies in vaccinated mice which is quite high as compared to subunit vaccines for SARS-CoV and MERS-CoV. These vaccines can be subjected to improvement by tailoring their immune profiles for disease specificity. Recently, a dual-targeting vaccine has been designed against Hepatitis B virus (HBV) using the target-specific dendritic cells and macrophages. As per the study in a chronic HBV mouse model 74, the vaccine reported the increased efficacy of viral clearance (Corbett et al., 2020). Hence, it has a tremendous inherent quality to serve as a vaccine. Recently, the ssRNA-lipid nanoparticle vaccine has been developed with self-amplification capability. Further research is needed for its dose rationalization and large scale clinical testing for it to be traded as a robust vaccine (Vazquez-Munoz and Lopez-Ribot, 2020).

## Barriers in Vaccine Development

Numerous hurdles are encountered in the process of vaccine development. Scale-up production of a particular vaccine requires a delicate balance between cost and quality. For e.g., in vector-based vaccines, the replication becomes defective due to the absence of all essential antigenic parts of the virus. The challenge lies in manufacturing these vaccines with high yield, removing the impurities, and economic production (Saha et al., 2020). The world-wide distribution also poses a challenge due to the lack of thermostability of the vaccines. Generally, vaccine requires refrigeration with temperature +2 and +8°C but some vaccines require even lower temperature which is difficult to fulfil in developing and also in developed countries. Due to this reason, lyophilized vaccines have gained attention to a greater extent. The outcome of the vaccine response is directly dependent on the route of administration (Iqbal Yatoo et al., 2020). The unique property of the vaccine is independent of the dosage, rather gets affected by the route of its entry into the host. For eg, a subunit vaccine by the name “Nasalflu Berna^®^” which was administered along with an adjuvant and an active heat-labile toxin (LT) from *Escherichia coli* was reported to cause facial paralysis. When administered through nasal route it generated Th1 immune response but this was not effective in suppressing the viral growth of SARS-CoV-2 in the lungs therefore was withdrawn from market early. Hence, commercialization of nasal vaccines becomes difficult. The need of the hour is development of a special delivery device for nasal vaccines. An alternative to this approach was the use of a nebulizer but it showed a reduction in virus titer after delivery. Therefore, delivery of vaccines *via* injections delivery is generally preferred for COVID-19 vaccines (Iqbal Yatoo et al., 2020); (Begum et al., 2020).

## Current Ongoing Approaches to Tackle SARS-CoV-2

**FIGURE 5:**
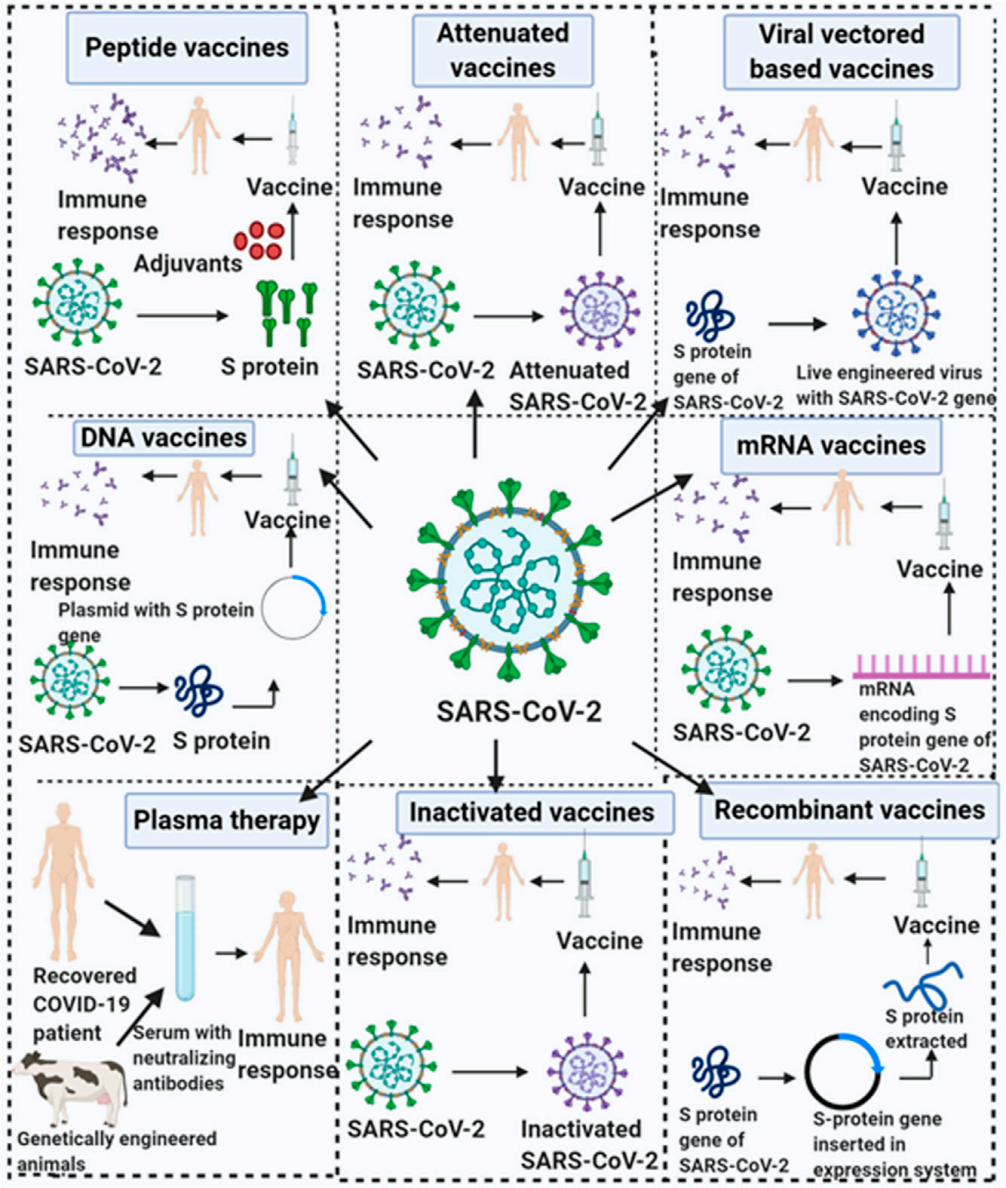
Overview of vaccine design and development.

Vaccine development is the culmination of complex, perilous, and expensive processes incorporating clinical development in the beginning, followed by process development and finally terminating with assay development. The vaccine race in India was evident by the triumphant sanctioning of BBVI52 or Covaxin on August 2020 and ZyCoV-D (Expected to release in 2021). Indigenous Covaxin was launched by Bharat Biotech International Limited (BBIL) in collaboration with the Indian Council of Medical Research (ICMR) after its final approval by DCGI on July 7, 2020. It was procured from a strain of SARS-CoV-2 isolated by the ICMR-National Institute of Virology, Pune. It initiated phase-1 clinical trials across the country in July 2020 and the results assured the safety of the vaccine after testing in 375 volunteers from 12 different sites. It successfully cleared the Phase 2 clinical trials which were conducted in 19 cities and in 10 different states of India seeking permission for its phase 3 trials on October 2, 2020. Zydus Cadila Healthcare Ltd traded the second vaccine under the label “ZyCoV-D” which went for the first phase of human clinical trials in July. After safely crossing the phase 1 trial across different countries it is currently undergoing phase 2 clinical trial. These two Indian vaccine candidates have undergone successfully *in vivo* clinical evaluation in rats, mice, rabbits with minimal known side effects, and reduced toxicity. This paved the way to start an early phase human trial under the approval of DCGI. Russia became the first nation to complete clinical assessments of the SARS-CoV-2 vaccine on humans, and the results have proved the effectiveness of the medication. It was denominated as “Sputnik V” in homage to the world’s first satellite, which is expected to launch in October 2020 by the Soviet Union. The formula was devised by the Gamaleya Institute in Moscow in coordination with the Russian defense ministry. It is based on a proven viral vectored vaccine against adenovirus that is administered in two doses each carrying an S-antigen of the novel SARS-CoV-2 that enters the human cells and produces an immune response. It emerged as the first registered vaccine against COVID-19 after successful completion of phase 1 and 2 trials and is currently undergoing Phase 3 trials in India after collaboration with Dr. Reddy’s lab with the distribution of 100 million doses of vaccine. The strategic collaborated approach of AstraZeneca and the University of Oxford proposed the global development and distribution of the University’s potential recombinant vaccine intended to prevent SARS-CoV-2 infection. The product was endorsed as a potential vaccine certified as ChAdOx1 COVID-19, by the Jenner Institute and Oxford Vaccine Group, at the University of Oxford. ChAdOx1 COVID-19 used a viral vector based on a weakened version of the adenovirus containing the genetic material of the SARS-CoV-2 S protein. After vaccination, S protein is produced, which primes the immune system to attack SARS-CoV-2 on the first encounter in the body. The recombinant adenovirus vector (ChAdOx1) was chosen to elicit a vigorous immune response by suppressing the viral replication after administration of a single oral dose that terminates ongoing infection in the vaccinated individual. Vaccines made from the ChAdOx1 virus were administered to more than 320 people starting from July 2020 to date and recorded as safe and well-tolerated, although they can cause temporary side effects such as flu-like symptoms and so on. It is one of the top eight vaccines being approved for human safety that is currently undergoing a Phase 2/3 trial. The Serum Institute of India would initiate delivery of the candidate vaccine at less than Rs 250/- per dose in India. The Pune-based institute partnered with Bill & Melinda Gates Foundation will offer up to 100 million doses, with the price capped at $3 for 92 low and middle-income countries including India. The outcome of its trial in monkeys revealed its protectiveness from viral pneumonia but cannot subside individuals from acquiring infections. Also, no significant difference in the amount of viral RNA was reported between vaccinated and unvaccinated monkeys. Thus, it was considered as a candidate that can decrease disease severity but not acquiring of infection. Novel Bnt162b1 vaccine was designed by Pfizer and BioNTech, incorporated nucleoside-modified RNA, that is required for the production of large amounts of antigen and robust immune responses is anticipated to be released by 2021. It also encodes the RBD of the SARS-CoV-2 S protein, a key target of virus-neutralizing Abs. Brazil, the nation with the second-highest number of coronavirus cases, reached an agreement to safeguard doses of the Oxford vaccine with AstraZeneca. Japan has signed up with Pfizer and BioNTech to provide 120 million doses of their vaccine and will be procured by October 2020. Clinical evaluation of its phase 1 and phase 2 trials generated a positive response. It is considered to be safe, efficacious in generating antibodies, and well-tolerated. Japan is also inking deals with Johnson & Johnson and Novavax to provide doses of their coronavirus vaccine. Newly, Sinovac Biotech has innovated a vaccine candidate that underwent interventional and observational pre-clinical evaluation *in vivo* and has flourished gracefully in producing IgG Abs. Another non-replicating virus vaccine, under the trademark “AZD1222”, was developed by Oxford University, UK was under Phase2/3 and in Phase 3 trials in South America and Brazil, respectively on July 20, 2020. It stimulated the production of neutralizing antibodies in the vaccinated host. The human trial of this vaccine candidate was paused for a few months after an individual developed neurological symptoms and adverse reactions. It resumed its trials in October after confirmation of its safety by MHRA in UK. CanSino Biologics of China thrived for a non-replicating COVID-19 vaccine endorsed as Ad5-nCoV is prepared to begin phase 3 clinical trials in Russia. Phase 1 trial started in April 2020 and generated a positive response. However, adverse reactions were found in very few individuals. Later on, it successfully cleared Phase 2. Siberian Vector Institute of Russia used the platform first developed for Ebola to develop the second Russian vaccine against COVID-19 which is under the Phase 1 trial. The UK government has signed a coronavirus vaccine deal with two drug giants GlaxoSmithkline (GSK) and Sanofi, to secure up to 60 million doses of an experimentally treated vaccine. The vaccine developed by Sanofi in partnership with GSK is based on the recombinant protein-based technology to produce a flu vaccine, as well as GSK's established pandemic technology. It is currently under phase 1/2 trials and is expected to move to Phase 3 by the end of 2020 if the results are positive. Also, BCG vaccines and Plant-based vaccines are developed by research institutes of Australia and Canada which are undergoing randomized trials. On December 5, Zydus Cadila has been approved by Government to initiate the phase three clinical trial of its biological therapy. The company has last month announced the successful completion of phase two clinical trial of its PEGylated Interferon alpha-2b biological therapy called “PegiHep TM”. The company has stated that the open-label, randomized, comparator-controlled study was conducted on 40 adult patients with moderate COVID-19 disease. Of these, 95% of subjects in the test who received a single dose of PegiHep TM along with the standard of care (SOC) became virus-free as assessed by RT-PCR on day 14, compared to only 68% those who only received SOC becoming RT-PCR negative. There are 10 vaccine candidates out of 42 which underwent phase 3 clinical trials involving about 30,000 participants as of October 2, 2020 (Table 9). Figure 5 represents an overview of vaccine design and development.

## Herbal Immunomodulation, Prophylaxis, and Treatment

**FIGURE 6:**
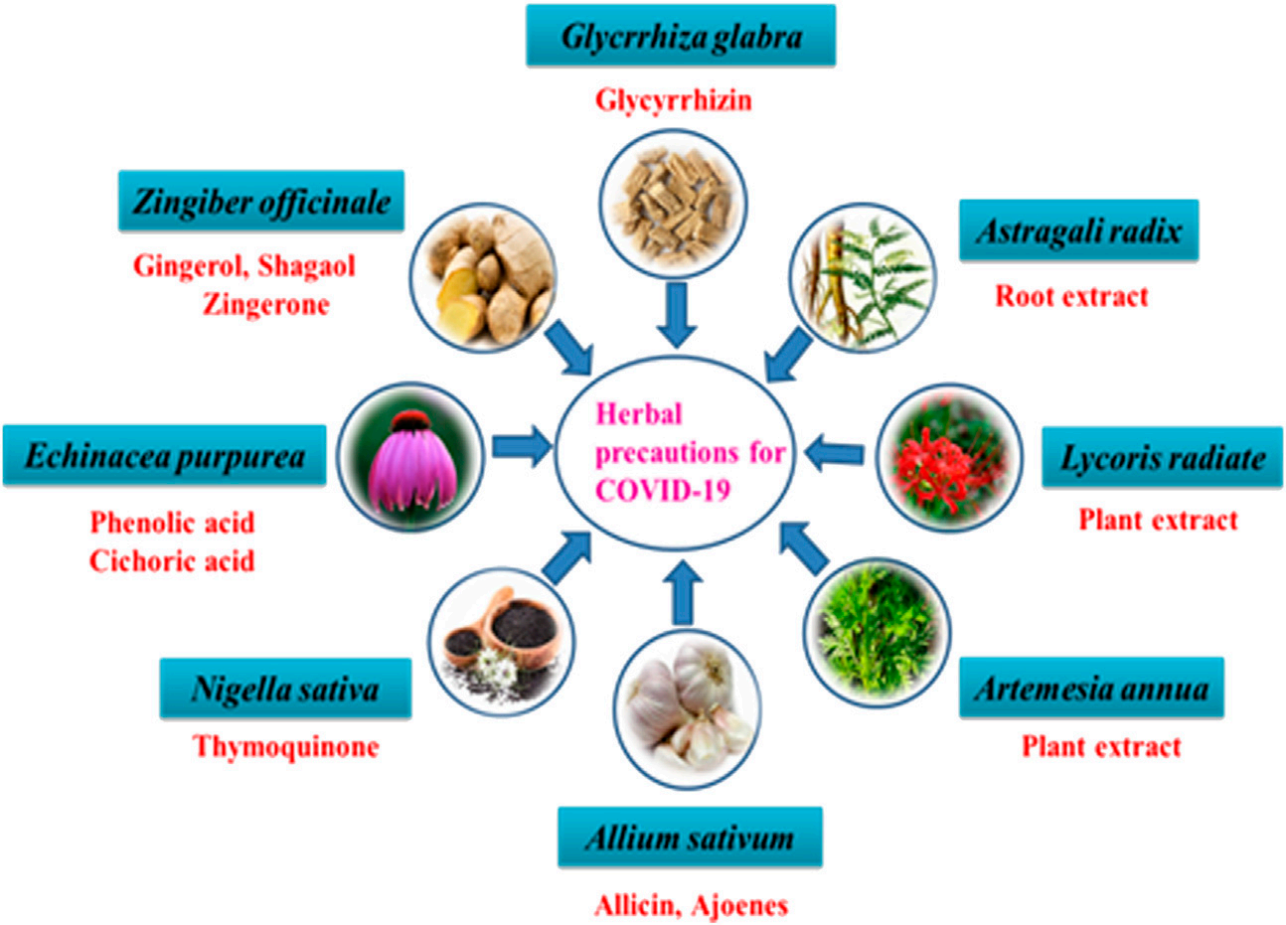
Herbal immunomodulatory remedies for the treatment of COVID-19.

The modern conventional vaccine development and production at the cost of time require strategic fast-paced trials, better infrastructure with large market potential, and also reporting potential health and safety issues. Therefore, it seems unrealistic to synthesize and develop new therapeutic molecules with improved pharmacokinetic profiles against this emerging pandemic to cure SARS-CoV-2 infection in all patients over a short period (Verma et al., 2020). In the 21st century, the incorporation of herbalism in the traditional medical system was found to yield cutting-edge cures in the field of antiviral research. Yupingfeng san (YPFS), traditional Chinese medicine (TCM) is a cocktail of three herbs Astragali radix (Astragalus), Fengfeng, and Atractylodes and has been convincingly proven to be highly potential to obtain a synergistic suppressive effect on the alleviated symptoms of obstructive lung disease (such as asthma, bronchitis, bronchiectasis) (Ang et al., 2020). Two TCM formulas like Sang Ju Yin and Yin Qiao San seemed to hold remedial measures for subsiding mild respiratory symptoms like fever, sore throat, cough and fatigue (Chen et al., 2020a). The potent inhibitory effect of plant extracts like Lycoris radiata (Red spider lily), Artemisia annua (Sweet Sagewort), Pyrrosia lingua (Felt Fern), and Lindera aggregata on SARS-CoV was observed by Li et al. (2005) (Jahan and Onay, 2020). Also, the active presence of phytoconstituents in these natural extracts such as TSL-1, Asecin, and consumption of herbal extracts of Allium sativum (Allicin, Ajoenes), Glycyrrhiza glabra (Glycyyhizin), Astragali radix (Astragalus), Zingiber officinale (Gingerol , Shagol, Zingerone), Echinacea purpurea (phenolic acid, Cichoric acid) act as natural immune system boosters (Tripathi et al., 2020) and elicits a strong immune response against the viral encounter. Reserpine confirms the potential therapeutic efficacy and slows down the rate of viral growth. Sambucus Formosana Nakai, a traditional medicinal herb is known to have strong antiinflammatory and antiviral properties. Allium sativum is well known as a functional food with immunomodulatory, antimicrobial, antiinflammatory, antimutagenic, antitumor properties (Donma and Donma, 2020). Turmeric is considered to be a traditional Ayurvedic medicine which contains curcumin, influencing multiple signaling pathways with anti-inflammatory, antioxidant, antimicrobial, hypoglycemic, wound healing, chemopreventive, chemosensitising and radiosensitising properties (Rastogi et al., 2020); (Gupta et al., 2020). The restrictive yield of SARS-CoV in Vero-E6 cells has been proved due to the use of Di-Kang injection, Lian-Hua-Qing-Wen capsule, and Fu-Fang-Lien-Pu pellets which are a combination of multiple herbs like Radix istidis (Ban-Lan-Gen), Fructus forsythia, Glycyrrhiza glabra (Licorice), and Lonicera kamtschatica etc. Some TCM formulae are fruitful in the treatment and prevention of COVID-19 but to a very limited extent as they specifically bind to major targets like S protein, ACE2, 3CLpro, PLpro, and RdRp activity. This therapy is a safe and viable alternative to antiviral drugs with no adverse side effects. Few of the tested herbal immunomodulatory drugs are saikosaponins, Myrecetin, Scutellarin, Artemisia annua (Sweet Sagewort), Isatis tinctoria (Woad), Pyrrosia lingua (Felt Fern), Tongue Fern), Atractylodis macrocephalae rhizome (Bai Zhu), etc (Panyod et al., 2020). There are hundreds of Chinese herbal immunomodulatory drugs that are anti-SARS-CoV in nature and demands immediate attention such as Quercetin, Andrographolide, Glycyrrhizin, Baicin, Patcholi alcohol, Luteolin, Hesperidin, Emodin, Tanshinone, Kaempferol, Curcumin, and Shikonin. The use of herbal-based treatment can be patient-specific and will be more beneficial and effective than other drugs (Divya et al., 2020). Because of the homology between the two viruses (SARS-CoV-2 and SARS-CoV), it is suspected to have the inherent potential that will be effective for suppressing COVID-19 symptoms (Boozari and Hosseinzadeh, 2020). Vitamin C may be effective against SARS-CoV-2 infection by validating its efficacy through clinical trial studies. Also, synergistic effect of vitamin C with polytherapy of Glycyrrhizin and curcumin actively regulates immune and inflammatory response associated with SARS-CoV infection (Bailly and Vergoten, 2020); (Chen et al., 2020b). There are uncountable food and herbs with immunomodulatory properties such as Aloe vera, Angelica gigas (Korean angelica), Astragalus membranaceus (Mongolian milkvetch), Ganoderma lucidum (lingzhi mushroom), Panax ginseng (ginseng), and Scutellaria baicalensis (Chinese skullcap), Azadiarachta indica (Neem), Ocimum tenuiflorum (Tulsi) (Mani et al., 2020); (Infusino et al., 2020). Figure 6 illustrates the herbal immunomodulatory remedies for the treatment of COVID-19.

## Conclusion and Future Perspectives

Contemplating the future of COVID-19, the development of a full-fledged vaccine may take a few months to a few years as a remedial measure for the on-going malady. Hence, the most effective way to counteract the present situation is to adapt oneself to the “new normal” taking into consideration social distancing (minimum six feet) and maintaining personal hygiene in an improved manner. This can effectively control viral transmission and reduce the number of incidences. Countermeasures to SARS-CoV-2 infection demands rapid testing, contact tracing, self-isolation of the infected individuals, and rapid antibody tests to identify asymptomatic cases. An attempt should be made by the government to uplift the safety guidelines and implement strict SOPs so that the livelihood of the general population return to its normalcy which will help to curtail the lethal spread of SARS-CoV-2 infection. As independent pieces of scientific evidence have revealed the long period survival of virion on copper, plastic, and steel for 4, 72, and 48 h respectively, hence proper sanitization driven disinfection of the public places, homes, and hospitals with 0.1% Dakin’s solution or 70% IPA, or 0.5% hydrogen peroxide should be imperative to mitigate the peak incidence including global death toll. Use of masks (N95) in association with personal protective equipment (PPE) should be mandatory for working individuals and the subjects displaying respiratory syndrome should be immediately given medical attention.

There are several vaccine candidates, currently undergoing multiple clinical trials to emerge as a prophylactic vaccine in the near future which will take a long time for approval and commercialization. Till then, repurposed investigational drugs are serving as a ray of hope to suppress the respiratory symptoms of the deadly COVID-19 pandemic. In the meantime, Convalescent plasma therapy and Mesenchymal stem cell therapy have gained substantial therapeutic attention as a remedial measure for the treatment of critically ill SARS-CoV-2 infected patients. Special attention should be provided in terms of therapeutics to the most vulnerable group, including healthcare individuals, children below 5 years, and old age people during this global pandemic. Pivotal implementation of telemedicine and online platforms for some months can contribute towards rapid prevention. COVID-19 disease directly targets the immune system which is a complex network of cells and proteins that defends our body against foreign invaders. Therefore, herbal therapeutics associated with immunomodulatory properties can be used as combinatorial prophylactic and adjunct therapeutics with chemical drugs to attain a positive synergistic strategy to combat this widespread pandemic of COVID-19. The foremost serious focus should be laid on several ways to boost the natural defense mechanism of the body i.e., by taking supplements with immune-boosting potentials such as neem, tulsi, astragalus, curcumin, and gingerone, and so forth. Globally ongoing clinical trials are being launched for validating the safety profile, efficacy, and therapeutic activity of these novel therapeutic herbal immunomodulatory agents. Detailed knowledge of SARS-CoV-2 molecular pathogenesis, clinical symptoms, and pathology are requisite to safeguard human life and reduce fatalities. Adapting to the “new normal” can lessen the economic burden on the world and prevent future pandemics. The rat race to develop efficacious therapeutics has united the whole world to combat COVID-19.

Recent reports from the clinical trial studies of four potential vaccine candidates have shown safe therapeutic efficacy against SARS-CoV-2 virion by eliciting an active immune response. Pfizer’s COVID-19 vaccine (BNT162Bb2) is found to be more than 90% effective based on initial trial results in 43,538 human volunteers with no safety issues. This vaccine is based on synthetic mRNA with a storage temperature of −70°C (ultra-cold) or below. India currently doesn’t have a system that can deliver such a vaccine, because the cold storage network of India’s universal immunization program currently handles temperatures in the range of 2–8 °C. Based on current projections, Pfizer expects to produce globally up to 50 million vaccine doses in 2020 and up to 1.3 billion doses in 2021. Also, another COVID-19 vaccine from Moderna has spurred a sell off in rival vaccine developers Pfizer, BioNTech, and AstraZeneca. Moderna’s vaccine candidate demonstrated an efficacy rate of 94.5% in a trial involving more than 30,000 infected patients. Its data revealed that side effects were generally short-lived and there were no significant safety concern, because no serious COVID-19 disease symptoms developed among trial participants. India’s SII also announced the completion of enrolment for phase 3 clinical trials. It was also observed that ICMR and SII have further collaborated for the clinical development of COVOVAX (Novavax) developed by Novavax, United States. Bharat Biotech is set to begin phase 3 trials of COVAXIN involving about 26,000 volunteers across India and this will be conducted in partnership with ICMR. It is the largest clinical trial conducted for a COVID-19 vaccine in India. These reports have installed a seed of faith that a promising vaccine will be available shortly and ease out the suffering that the world is facing today due to this deadly pandemic. However and whatever be the situation humanity has always prevailed under affliction and the way scientists all over the world have united to find a permanent treatment for COVID-19 sooner than later the cure will be assured until then personal implementation of the protective measure remains inevitable.
